# Longer epidermal cells underlie a quantitative source of variation in wheat flag leaf size

**DOI:** 10.1111/nph.18676

**Published:** 2022-12-28

**Authors:** Camila M. Zanella, Marilena Rotondo, Charlie McCormick‐Barnes, Greg Mellers, Beatrice Corsi, Simon Berry, Giulia Ciccone, Rob Day, Michele Faralli, Alexander Galle, Keith A. Gardner, John Jacobs, Eric S. Ober, Ana Sánchez del Rio, Jeroen Van Rie, Tracy Lawson, James Cockram

**Affiliations:** ^1^ NIAB 93 Lawrence Weaver Road Cambridge CB3 0LE UK; ^2^ University of Messina Messina 98122 Italy; ^3^ University of Manchester Manchester M13 9PL UK; ^4^ Limagrain UK Ltd Market Rasen LN7 6DT UK; ^5^ School of Biological Sciences University of Essex Colchester CO4 3SQ UK; ^6^ BASF Belgium Coordination Center (BBCC) – Innovation Center Ghent Technologiepark‐Zwijnaarde 101 9052 Ghent Belgium

**Keywords:** flag leaf morphology, haplotype analysis, maximum stomatal conductance (*G*_smax_), multifounder advanced generation intercross population, quantitative trait variation, wheat (*Triticum aestivum* L.)

## Abstract

- The wheat flag leaf is the main contributor of photosynthetic assimilates to developing grains. Understanding how canopy architecture strategies affect source strength and yield will aid improved crop design.
- We used an eight‐founder population to investigate the genetic architecture of flag leaf area, length, width and angle in European wheat. For the strongest genetic locus identified, we subsequently created a near‐isogenic line (NIL) pair for more detailed investigation across seven test environments.
- Genetic control of traits investigated was highly polygenic, with colocalisation of replicated quantitative trait loci (QTL) for one or more traits identifying 24 loci. For QTL *QFll.niab‐5A.1* (*FLL5A*), development of a NIL pair found the *FLL5A+* allele commonly conferred a *c.* 7% increase in flag and second leaf length and a more erect leaf angle, resulting in higher flag and/or second leaf area. Increased *FLL5A‐*mediated flag leaf length was associated with: (1) longer pavement cells and (2) larger stomata at lower density, with a trend for decreased maximum stomatal conductance (*G*_smax_) per unit leaf area.
- For *FLL5A*, cell size rather than number predominantly determined leaf length. The observed trade‐offs between leaf size and stomatal morphology highlight the need for future studies to consider these traits at the whole‐leaf level.

## Introduction

Bread wheat (*Triticum aestivum* L.) underpins human food security across the world. Wheat grain yield is determined by many constitutive traits across the plant lifecycle (White *et al*., 2021), representing the product of cumulative photosynthetic activity across the growing season combined with the capacity of the developing grain to store these products (Zelitch, 1982). The uppermost leaf of a wheat plant, the flag leaf, plays a major role in supplying the developing grain with photoassimilates after anthesis (Araus & Tapia, 1987; Sharma *et al*., 2003). Therefore, flag leaf morphological traits, such as area, length and width, represent important factors determining overall plant structure, capacity to intercept light and final yield potential. Additionally, leaf angle influences the efficiency of light interception and overall canopy structure, which influencing photosynthetic efficiency. Cultivars with erect leaves generally have increased light interception due to greater light penetration through the canopy, leading to improved photosynthetic capacity, enhanced grain filling rate (Austin *et al*., 1976; Richards *et al*., 2019) and better heat tolerance (Hunt *et al*., 2018). Indeed, erect leaves represent a component of an ideal wheat ideotype (Donald, 1968). Therefore, precise understanding of the genetic control of ‘source strength’ traits such as leaf morphology and angle will provide tools to further explore and refine specific phenotypic combinations to help support increased grain yield.

Throughout the vegetative stages of wheat growth, leaf primordia develop at the periphery of the shoot apical meristem. Their formation is controlled by five principal stages: initiation, general cell division, transition, cell expansion and meristem division (Gonzalez *et al*., 2012). The leaf primordia then develop into flat leaves after tissue differentiation along several planes, including the adaxial–abaxial axis that determines the top and bottom sides of the leaf, respectively. Such stages ultimately govern final leaf size, and heritable variation in the genes controlling these processes can result in changes in leaf morphology. To date, just one gene controlling wheat flag leaf traits has been map‐based cloned: an artificial mutant of the gene *TaSPL8*, encoding a SQUAMOSA PROMOTER BINDING‐LIKE (SPL) protein (Liu *et al*., 2019). Mutation of *TaSPL8* results in erect leaves due to loss of the lamina joint connecting the leaf blade to the leaf sheath, possibly *via* disruption of the auxin signalling and brassinosteroid biosynthesis pathways (Liu *et al*., 2019). However, leaf size mutants have been map‐based cloned in the related crop species rice (*Oryza sativa* L.) and maize (*Zea mays* L.). Several of these genes are involved in auxin production or transport: The rice *narrow leaf 1* (*nal1*) mutant is due to mutation of a gene thought to affect polar cell‐to‐cell auxin transport and vascular tissue differentiation and patterning (Qi *et al*., 2008), while the narrow and curly leaf phenotype of *nal7*, which also includes the presence of smaller bulliform cells (structural cells present on the adaxial epidermis that typically control leaf rolling in response to water stress), is due to a mutation in *YUCCA8* (*YUC8*) involved in auxin biosynthesis (Fujino *et al*., 2008). Similarly, *WUSCHEL‐related homoeobox 3* (*WOX3*) genes underlie leaf size mutants in both maize (*narrow sheath1* (*ns1*) and *ns2*) (Nardmann *et al*., 2004) and rice (*narrow leaf2* (*nl2*) and *nl3*) (Sung‐Hwan *et al*., 2013). In both species, these *WOX3* genes play a role in the recruitment of founder cells for the margin development of lateral organ primordia, including the leaves. Their mutation affects several phenotypes, including the production of narrow leaves. Lastly, the rice mutant *narrow and rolled leaf 1* (*nrl1*) encodes the cellular synthase‐like D4 gene *OsCslD4*, whose mutation results in smaller bulliform cells – likely due to defects in cell wall biosynthesis (Hu *et al*., 2010). In rice, leaf angle is controlled by components of the brassinosteroid hormone synthesis and signalling pathways, as well as the interaction of several helix–loop–helix (HLH) transcription factors (summarised by Guo *et al*., 2021).

While *TaSPL8* represents the only map‐based cloned wheat leaf morphology gene, genetic studies have identified numerous QTL for flag leaf size and angle (e.g. Xue *et al*., 2013; Fan *et al*., 2015; Wu *et al*., 2016; Yang *et al*., 2016; Hussain *et al*., 2017; K. Liu *et al*., 2018; Y. Liu *et al*., 2018; Marzougui, 2019; Stadlmeier *et al*., 2019; Jin *et al*., 2020; Ma *et al*., 2020; Yan *et al*., 2020; Scott *et al*., 2021; Tu *et al*., 2021). To date, the most finely mapped natural variant controlling flag leaf morphology is the genetic locus *QFlw.nau‐5A.1* controlling flag leaf width, mapped to a 0.2 cM genetic interval on the long arm of chromosome 5A (Xue *et al*., 2013). Despite the number of flag leaf QTL identified to date, only a small proportion is stable across environments, indicating interaction with the environment plays a notable role in final leaf morphology. Investigating the genetics of wheat flag leaf traits has predominantly been undertaken using bi‐parental populations. More recently, multifounder populations (reviewed by Cockram & Mackay, 2018; Scott *et al*., 2020) have been developed in crop species, including wheat. One advantage of such populations is that they typically capture higher levels of genetic variation within a unified genetic platform.

Here, we used an eight‐founder wheat multiparent advanced generation intercross (MAGIC) population to define the genetic control of five flag leaf morphological traits (length, width, length : width ratio, area and angle). We then developed a near‐isogenic line (NIL) pair for one of the identified flag leaf QTL (*QFll.niab.5A.1*), allowing the effect of contrasting alleles at this locus to be further explored phenotypically within genetic backgrounds that were otherwise *c*. 98% identical. We found increased flag leaf length was associated with longer epidermal pavement cell size, indicating cell length rather than cell number predominantly underlies the effect of allelic variation at this locus. Furthermore, the long leaf allele was associated with longer, but less dense, stomata, with this trade‐off resulting in a trend for decreased maximum stomatal conductance (*G*_smax_) per unit leaf area. Collectively, our results provide physiological insights into the genetic control of wheat flag leaf morphology, providing entry points to further explore how contrasting canopy architecture and associated epidermal cell patterning strategies impact plant performance under different environments.

## Materials and Methods

### Wheat germplasm, genotypic data and field trials

The ‘NIAB Elite MAGIC’ wheat (*T. aestivum* L.) population is described previously (Mackay *et al*., 2014). Briefly, it was constructed by intercrossing eight founders (cvs Alchemy, Brompton, Claire, Hereward, Rialto, Robigus, Soissons and Xi19) over three generations followed by selfing to generate recombinant inbred lines (RILs). Genotypic data were generated previously by Mackay *et al*. (2014) using the wheat 90K single‐nucleotide polymorphism (SNP) array (Wang *et al*., 2014), with further manual curation (Gardner *et al*., 2016). The population was grown in four field trials in Cambridgeshire, UK. Each trial was autumn‐sown, reaching maturity the following summer. The 2016 and 2017 season trials followed a p‐rep design, while each RIL in the 2018 and 2019 trials were present in two reps (Supporting Information Methods S1). For all trials, each entry was grown as a 2 × 6 m plot consisting of six 6 m rows, grown using standard winter wheat agronomic packages (Table S1). For details of trial site locations, planting dates and trial designs, see Table S2.

### Leaf phenotyping

Fully expanded flag leaves were sampled from *c*. 150 plots per day. Sampling was conducted when the plots were between Zadoks growth stage (GS) 59 (ear emergence complete) and GS69 (anthesis complete). Leaves were placed into sealable plastic bags, misted with water and stored at 4°C until imaged later the same day using WinDias Leaf Image Analysis System (Delta‐T, Burwell, UK). A size standard was included in each image to convert pixel count to measurement in mm. At NIAB16, six flag leaves were harvested per plot, while at NIAB17, NIAB18 and NIAB19, 12 flag leaves were harvested per plot. Using the resulting image files, flag leaf length (mm), width (mm) and area (mm^2^) were measured using ImageJ (Schneider *et al*., 2012), and flag leaf length : width ratio was subsequently calculated. Flag leaf angle was assessed visually for all plots on a single day. The angle assessed at a plot level was that of the mid‐region of the flag leaf relative to the vertical plane using a 7‐point scale, where 0 = vertical upwards and 6 = vertical downwards. Half points on the scale were also scored, and the resulting data were doubled before downstream trials analyses resulting in a 0–12 scale.

### Trials analysis, phenotypic correlations and trait heritability

The raw phenotypic data were spatially analysed by fitting two‐dimensional P‐spline mixed models implemented in the R package spats (Rodríguez‐Álvarez *et al*., 2018). Spatial effects were modelled on a row and column basis using the separation of anisotropic penalties algorithm introduced by Rodríguez‐Álvarez *et al*. (2015), with the number of segments set to the respective number of rows and columns from the experimental design. For each trial, row and column were modelled as random effects. The best linear unbiased predictors (BLUPs) of genotype effects were predicted from a fitted spats object. Generalised heritability proposed by Cullis *et al*. (2006) and Oakey *et al*. (2006) was estimated using the spats model (getHeritability function). The BLUPs were then used as genotypic values to perform the QTL mapping. Phenotypic correlations were estimated between BLUPs using Pearson's correlation coefficients and paired Wilcoxon signed‐rank test using the hmisc package (Harrell, 2019) and plotted using the package corrplot (Wei & Simko, 2017), and statistically significant associations denoted: ***, *P* < 0.001; **, *P* < 0.01; *, *P* < 0.05.

### Genetic analyses

Following our published analysis pipeline (Downie *et al*., 2018; Corsi *et al*., 2021), genetic analysis was carried out using four approaches: (1) Identity‐by‐state single‐marker analysis: using bi‐allelic SNP classes and undertaken in R/lme4 (Bates *et al*., 2015). (2) Identity by descent: regression against haplotype probability estimates calculated using the function ‘mpprob’ in R/mpmap (Huang & George, 2011) implemented in R/qtl (Broman *et al*., 2003) with a threshold of 0.5. (3) Interval mapping (IM): undertaken in R/mpmap using haplotype probability estimates calculated using R/mpmap. (4) Composite interval mapping (CIM): conducted using R/mpmap with 5 (CIM‐cov5) or 10 (CIM‐cov10) covariates using haplotype probability estimates calculated using R/mpmap. For further details, see Methods S1.

### Bioinformatic analyses

Single‐nucleotide polymorphisms from the 90K array were anchored to the wheat reference genome assembly (cv Chinese Spring 42 RefSeq v.1.0; IWGSC *et al*., 2018) *via* Blastn (Altschul *et al*., 1990), as described by Corsi *et al*. (2021). To investigate how haplotypes at our chromosome 5A QTL translated into wider germplasm, a panel of 403 European wheat cultivars parsed from those available at https://www.niab.com/research/agricultural‐crop‐research/resources was used to determine haploblocks across chromosome 5A using Haploview v.4.2 (Barrett *et al*., 2005). Within the manually curated haploblock spanning the peak SNP from the flag leaf length meta‐analysis, haplotypes were extracted. Genotype calls at the SNPs defining the haploblock were also determined in the 15 *T. aestivum* varieties for which genome assemblies are available (IWGSC *et al*., 2018; Walkowiak *et al*., 2020) *via* Blastn. Relationships between haplotypes were determined using hierarchical clustering analysis (Euclidean distance) in Rstudio (RStudio Team, 2020). To identify candidate genes, cloned genes controlling leaf size or angle in related cereal species (rice, maize and barley) were identified *via* literature search, and their coding regions (CDS) used as Blastn queries against the wheat reference genome.

### NIL germplasm development

Analysis of the 90K array SNP data surrounding the target QTL on chromosome 5A allowed identification of RILs heterozygous across the QTL as described in Methods S1 and resulted in prioritisation of RIL MEL_018_2 for NIL pair development. A Kompetitive Allele‐Specific PCR (KASP) marker was designed for SNP *BS00062996_51*, shown to be heterozygous in MEL_018_2 at the F_5_ generation. DNA sequence flanking the SNP was used to design KASP primers using PolyMarker (Ramirez‐Gonzalez *et al*., 2015; Table S3) and validated as described in Methods S1. Ten sibling F_5_ seeds for RIL MEL_018_2 were grown, and DNA extracted from each individual for subsequent KASP genotyping. Selected F_5_ individuals identified as homozygous C:C or T:T were retained, polythene bags attached to each of the developing ears before anthesis, and the resulting selfed F_6_ seed harvested. These F_6_ germplasm stocks represented the initial seed stock for the *QFll.niab‐5A.1* NIL pair.

### NIL phenotyping and analysis

The NIL pair was grown in five autumn‐sown UK field trials (Table S4) and phenotyped the following summer. In all field trials, the flag leaf (leaf‐1), as well as the two subsequent leaves down the stem (leaf‐2 and leaf‐3), length and width were measured using a ruler in mm. Additionally, for trials NIAB 2021 and LIM 2021, manual measurements from 30 main tillers per plot were recorded for leaf‐1 to leaf‐5, as well as all internode distances, interleaf distances, ear length, spikelet number and flag leaf angle. The NIL pair was also assessed for leaf morphology under two glasshouse experiments (GH 2018 and GH 2021), grown as described in Methods S1. The length and width of leaf‐1, leaf‐2 and leaf‐3 from the main tiller of each plant were measured in mm using a ruler at GS69 (anthesis complete). All NIL data were checked for normality and homoscedasticity through visual assessment of the distribution and residuals vs fitted values. A two‐way analysis of variance (ANOVA) was carried out for flag leaf width, length and area (calculated as leaf area = leaf width × leaf length × 0.858, as described in Gioia *et al*., 2015) with Allele and Leaf as factors. Differences between NIL for the same leaf were assessed *via* one‐way ANOVA. Statistical analyses were carried out using Rstudio. Violin plots and bar charts were produced in Rstudio using ggplot2 (Wickham, 2016). In experiment GH 2021, leaf emergence was also measured in every 1–3 d after the completion of vernalisation treatment, scored to the nearest quarter of a leaf.

Flag leaf epidermal pavement cell length was measured *via* scanning electron microscopy (SEM) in the NIAB 2021 trial, using 15 flag leaves harvested from *FLL5A+* plot 49 and from *FLL5A−* plot 48. Leaves sampled were of average length for each plot: 17.0 cm for *FLL5A+* and 15.8 cm for *FLL5A−*. Sections of 8 × 8 mm sampled from the centre of each leaf were used for SEM, as described in Methods S1. Three SEM images per leaf were taken, and the lengths of epidermal cells adjacent to stomatal tracks measured using ImageJ (Schneider *et al*., 2012), with typically 30–50 cells measured per image. Density plots of cell size data were undertaken using the package ggstance in Rstudio. To measure flag leaf pavement cell size in glasshouse experiment GH 2021, three leaf size classes were sampled. Class 1 (termed ‘small’): 3–5 flag leaves of mean length for that observed in the *FLL5A−* plots were sampled in the *FLL5A*− and *FLL5A+* plots (25.2 ± 0.5 cm). Class 2 (termed ‘large’): 3–4 flag leaves of mean length for that observed in the *FLL5A+* plots were sampled in the *FLL5A−* and *FLL5A+* plots (30.8 ± 0.5 cm). Class 3 (termed ‘largest’): 4–5 flag leaves of a size larger than the observed mean for *FLL5A+* plots (32.5 ± 0.5 cm) were sampled in the *FLL5A−* and *FLL5A+* plots. Imprints of the lower epidermis at the centre of each leaf were made as described by Wilson *et al*. (1986), and pavement cell imaging undertaken as described previously, with an average of 128 cells per leaf measured. For trial NIAB 2022, imprints were taken from the base, middle and tip of the upper (adaxial) side of the leaf blade in flag leaves of equal length (11.3 ± 0.3 cm) and width (1.6 ± 0.2 mm) for *FLL5A+* (plot 66) and *FLL5A−* (plot 55).

Stomata size (guard cell length), stomata density mm^−2^, stomata row number mm^−2^ and stomata number per stomatal row mm^−2^ were measured from the SEM (NIAB 2021) and imprint (GH 2021, NIAB 2022) images, using the methods described previously. Maximum stomatal conductance (*G*_smax_) was estimated as described previously (Franks & Farquhar, 2001).

## Results

### MAGIC founders employ differing flag leaf morphologies to capture sunlight

**Fig. 1:**
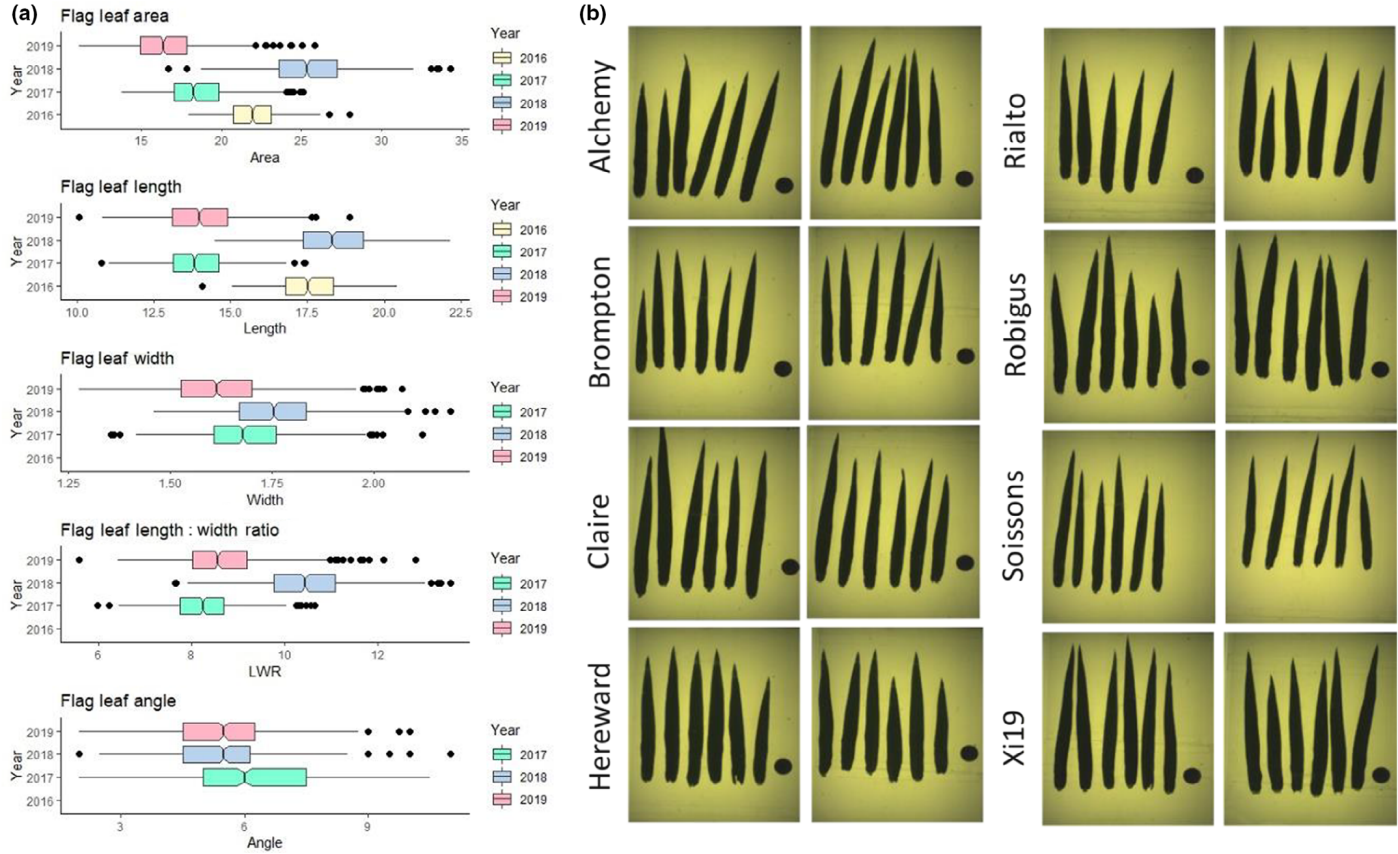
Wheat (*Triticum aestivum* L.) MAGIC founder flag leaf phenotypes. (a) Boxplots of flag leaf phenotypes from trials NIAB16, NIAB17, NIAB18 and NIAB19. The horizontal lines denote the median, boxes indicate the lower (25%) and upper (75%) quartiles, whiskers indicate the ranges of the minimum and maximum values, and dots predicted outliers. Flag leaf width from NIAB16 was not used, as explained in the Results section. (b) Examples of MAGIC founder flag leaf images from trial NIAB18. Six leaves from plot replicate‐1 and six leaves from plot replicate‐2 shown for each founder. Descriptive flag leaf ideotypes for each founder are listed here, including angle: Alchemy: long leaf with intermediate width resulting in an intermediate area combined with an erect angle. Brompton: short length with intermediate width resulting in a low area combined with intermediate angle. Claire: longest length with intermediate width resulting in the highest area combined with intermediate angle. Hereward: medium length with the widest leaf resulting in high area combined with erect angle. Rialto: short length with wide leaf resulting in intermediate area combined with the most erect angle. Robigus: medium length with wide lead resulting in intermediate area combined with intermediate angle. Soissons: medium length with the narrowest leaf resulting in low area combined with lax angle. Xi19: long length with intermediate width resulting in intermediate area with the most lax angle.

Five flag leaf traits (length, width, area, length : width ratio and angle) were measured in four UK field trials, and BLUPs calculated for each MAGIC founder and RIL (Fig. 1a; Table S5). Trends in flag leaf sizes were observed for the eight founders (Table S6), with examples of founder flag leaf images and main tillers shown in Figs 1(b) and S1, respectively. Flag leaves for Claire were both the longest (mean = 18.1 cm) and the largest area (mean = 24.3 cm^2^) of all founders. Brompton and Rialto consistently had the shortest leaves. However, Rialto had the second widest flag leaf (mean = 1.86 cm) and Brompton the second narrowest (mean = 1.62 cm). While Brompton flag leaf area was the lowest of all the founders (mean = 19.5 cm^2^), Rialto had a mid‐ranking area (mean = 21.7 cm^2^). Hereward, Rialto and Robigus had the widest flag leaves (1.89, 1.86 and 1.84 cm, respectively), with Soissons being by far the narrowest (mean = 1.45 cm). Flag leaf angle was the most erect in Rialto (mean = 4.1) and most lax in Xi19, Soissons and Brompton (mean = 7.8, 6.6 and 6.3, respectively). Together, these data indicate the eight founders employ differing combinations of flag leaf size and angle traits to capture sunlight at the top of the wheat canopy.

### Trait correlations in the MAGIC RILs show leaf width is negatively correlated with angle

**Fig. 2:**
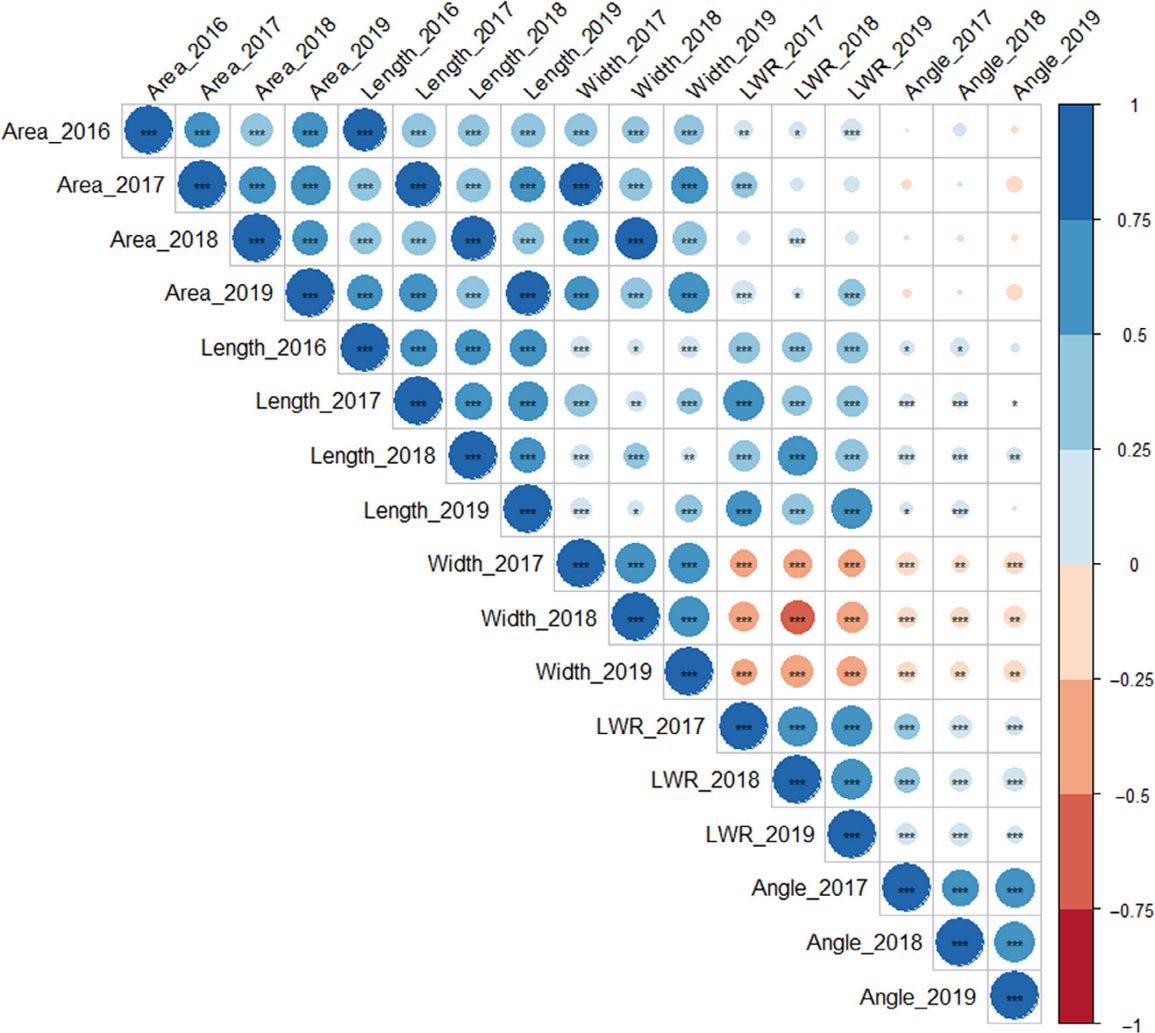
Correlations between the five flag leaf traits measured in the winter wheat (*Triticum aestivum* L.) ‘NIAB Elite MAGIC’ population grown in field trials undertaken in the UK in harvest seasons 2016, 2017, 2018 and 2019. Trait abbreviations: Flag leaf length (Length), flag leaf width (Width), flag leaf area (Area), flag leaf length : width ratio (LWR), flag leaf angle (Angle). Significant correlations at *, *P =* 0.05; **, *P =* 0.01; ***, *P =* 0.001 determined by paired Wilcoxon signed‐rank test are indicated.

Analysis of flag leaf width data from 2016 found strong spatial patterns in the first 24 columns of the trial. After their removal, heritability remained low (*h* ^2^ = 0.37), and so this dataset was excluded from further analysis. Of the remaining 17 trait per year combinations, high heritability was observed (mean *h* ^2^ = 0.78), ranging from *h* ^2^ = 0.91 for flag leaf width in 2018 to *h* ^2^ = 0.56 for flag leaf area in 2016 (Table 1). For all traits, transgressive segregation (where the observed RIL phenotypic variation exceeded that of the eight founders) was observed in both directions, most notably for flag leaf length in 2016 and 2018 (Fig. S2). Strong positive correlations were observed between flag leaf length and area within all years (*R* ≥ 0.57, *P <* 0.001; Fig. 2). Flag leaf width was positively correlated with area (*R* ≥ 0.53, *P <* 0.001) and to a much lesser extent with length (*R* ≥ 0.26, *P <* 0.001), indicating genetic control of width may to some extent be independent to that of length. Interestingly, while angle was significantly positively correlated with length within and between all environments, it also showed a negative correlation with leaf width (*R ≤* −0.18, *P ≥* 0.002).

**Table 1:** Heritabilities for the five flag leaf traits measured on the NIAB Elite MAGIC population in four test environments (harvest seasons 2016, 2017, 2018 and 2019).

Flag leaf trait per year	Heritability (*h* ^2^)
Area 2016	0.56
Area 2017	0.72
Area 2018	0.81
Area 2019	0.81
Length 2016	0.69
Length 2017	0.73
Length 2018	0.81
Length 2019	0.81
Width 2016	0.37
Width 2017	0.81
Width 2018	0.91
Width 2019	0.89
Length : width ratio 2017	0.81
Length : width ratio 2018	0.90
Length : width ratio 2019	0.87
Angle 2017	0.87
Angle 2018	0.89
Angle 2019	0.89

### Control of flag leaf morphology is highly polygenic

**Fig. 3:**
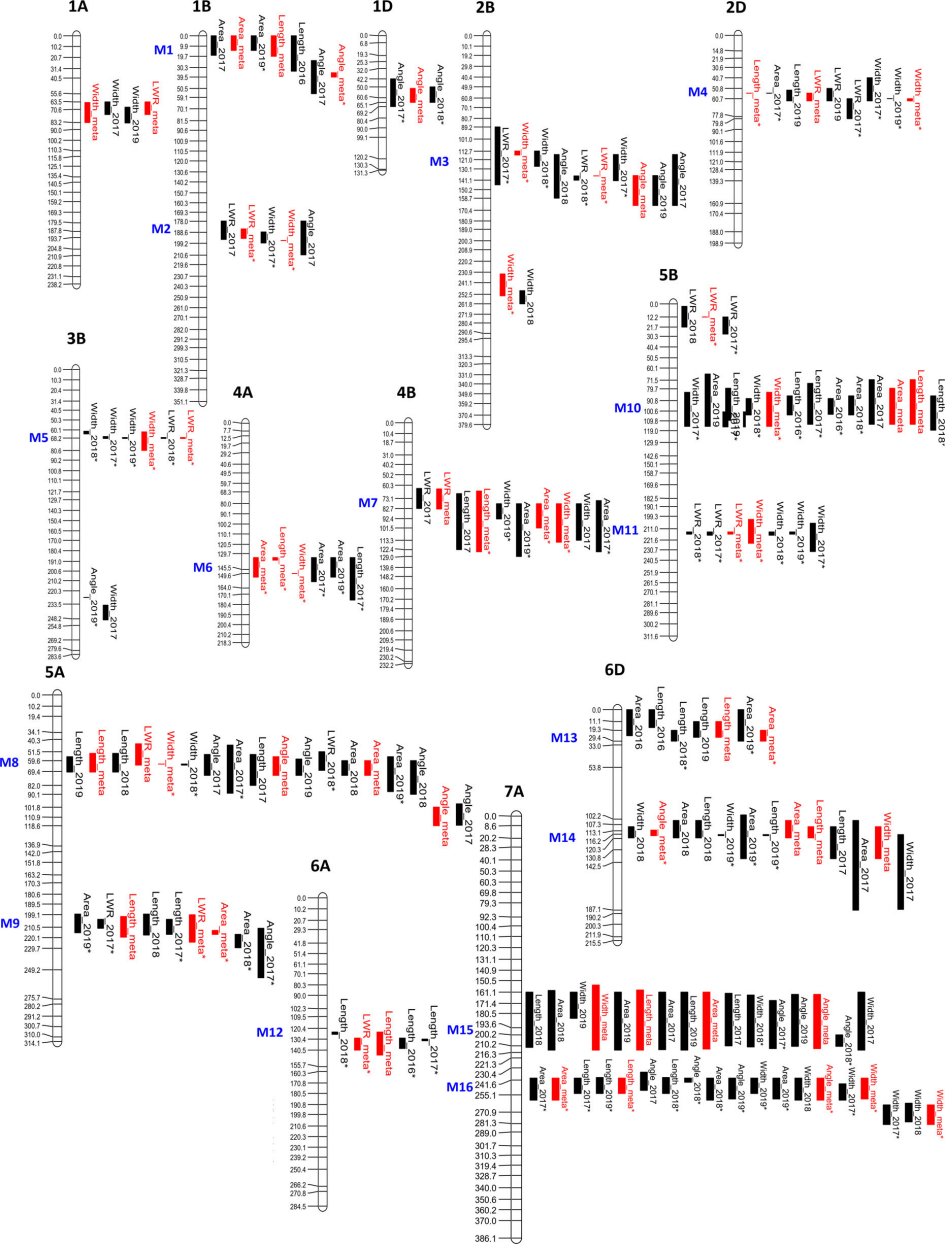
Quantitative trait loci (QTL) identified for flag leaf traits in the ‘NIAB Elite MAGIC’ wheat (*Triticum aestivum* L.) population. Trials were conducted in harvest season 2016, 2017, 2018 and 2019. Genetic intervals for QTL and meta‐QTL are indicated in black or red, respectively. QTL identified using identity‐by‐state (IBS) and/or identity‐by‐descent (IBD) mapping approaches only, are indicated with an asterisk. The locations of multitrait QTL (M; genetic loci containing replicated QTL for two or more traits) are indicated. For illustration purposes, only a subset of the genetic markers in the MAGIC genetic map (Gardner *et al*., 2016) are shown here; units shown are centimorgans (cM).

Flag leaf traits were found to be controlled by numerous genetic loci of relatively small effect, with the phenotypic variance explained ranging from 3.6% (length : width ratio, *QFlr.niab‐5B.1*) to 10.2% (length : width ratio, *QFlr.niab‐2D.1*; mean = 5.4%; Table S7). Fifty‐seven QTL were identified for the five flag leaf traits investigated, located across 13 of the 21 wheat chromosomes: 12 QTL for length, 15 for width, 11 for area, 9 for length : width ratio and 10 for angle (Fig. 3; Table S7). Of these, 38 (67%) were replicated in two or more environments. The number of replicated QTL was proportionately higher for flag leaf area (91%) than for the remaining four traits (ranging from 44% for length : width ratio to 73% for width). Of these 57 QTL, 50 colocated with QTL for one or more additional flag leaf traits and are termed here ‘meta‐QTL’. Accordingly, 24 distinct genetic loci were resolved, which included 16 meta‐QTL (Fig. 3; Table S7). Unsurprisingly given flag leaf area is predominantly defined by leaf length and width, all eight flag leaf length QTL colocated with QTL for area, and the remaining four area QTL colocated with genetic loci controlling width. Meta‐QTL *QMFl.niab‐5A.1* was notable in being the only locus with colocating QTL for all five flag leaf phenotypes (Fig. 3). Where calculated *via* IM analysis, the percentage of the phenotypic variance explained by *QMFl.niab‐5A.1* was up to 6% for length, 5.2% for area and 7.4% for angle. Meta‐QTL *QMfl.niab‐2D.1* controlling flag leaf area (identified in the 2017 season trial and meta‐analysis), width (trial 2017) and angle (trial 2018) colocated with the photoperiod response locus *Photoperiod‐D1* (Table S7; Bentley *et al*., 2013), and so likely represented pleiotropic effects of this major gene controlling crop development. Although alleles controlling semi‐dwarfing phenotype at the *Reduced height‐B1* (*Rht‐B1*) and *Rht‐D1* genes on chromosomes 4B and 4D were known to segregate in the population, no flag leaf traits were identified at these locations. To establish *a priori* candidate genes, we identified 28 cloned genes controlling leaf size or angle in rice, maize and barley and determined their wheat homologues (Table S8). Comparing their positions with our MAGIC QTL found candidates to be present at three relevant locations: *QAng.niab‐1D.1* and meta‐QTL on chromosomes 5A (M8) and 7A (M15). For further details, see Fig. S3 and Methods S1.

### Genetic locus FLL5A controls increased length and area in the upper canopy

**Fig. 4:**
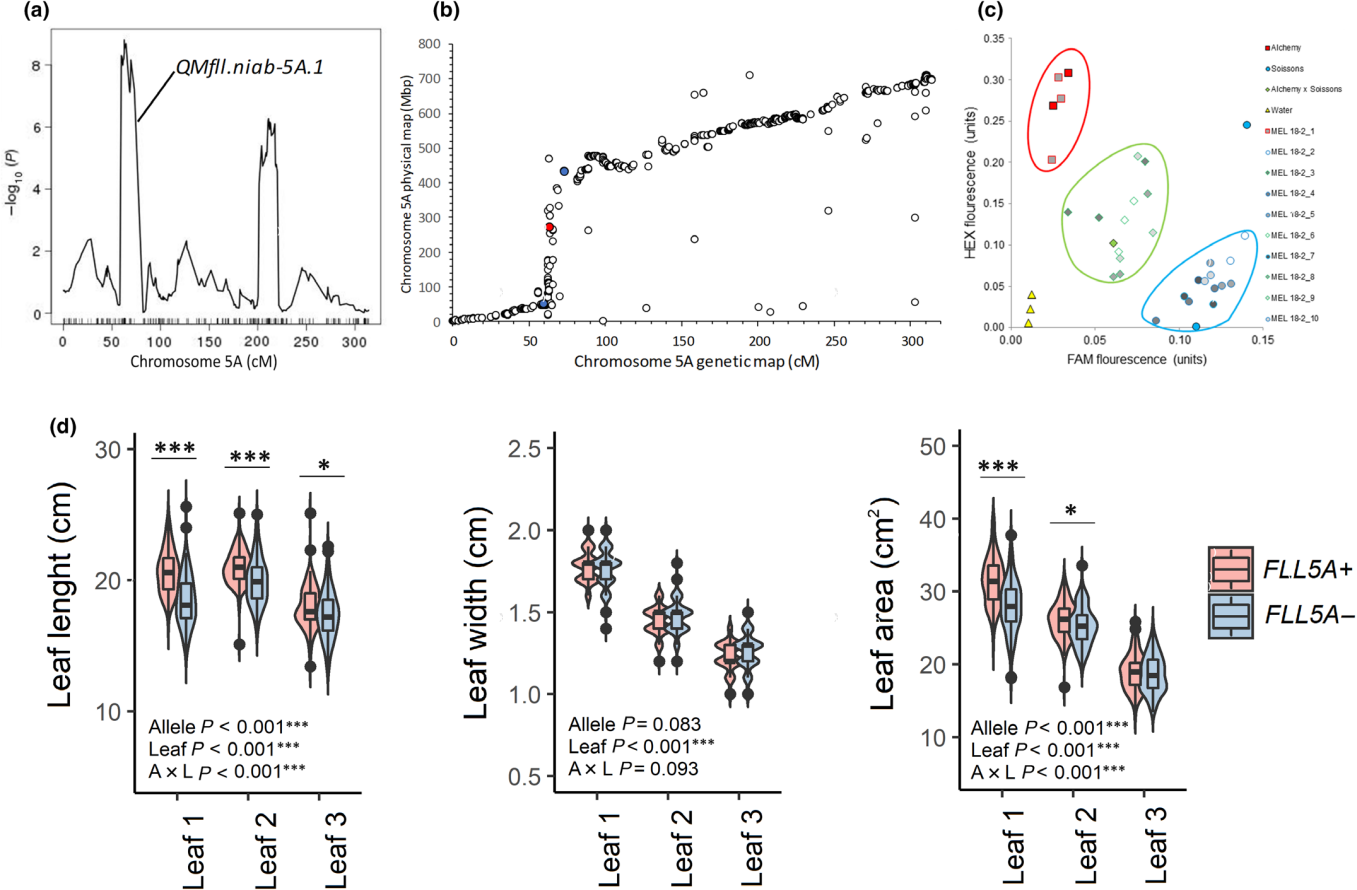
Development and assessment of a near‐isogenic line (NIL) pair for QTL *QFll.niab‐5A.1*. (a) Results of meta‐QTL analysis for flag leaf length using genetic analysis method CIM‐cov10, showing chromosome 5A QTL *QFll.niab‐5A.1*. (b) Comparison of genetic (Gardner *et al*., 2016) vs physical (IWGSC *et al*., 2018) maps shows the QTL interval is in the region of low genetic recombination spanning the chromosome 5A centromere. The *QFll.niab‐5A.1* peak marker is shown in red, and the left and right flanking markers are shown in blue. (c) The use of a codominant Kompetitive Allele‐Specific PCR marker for single‐nucleotide polymorphism (SNP) *BS00062996_51* to screen 10 F_5_ sibling individuals from MAGIC recombinant inbred line (RIL) MEL_018_2. This RIL was heterozygous across *QFll.niab‐5A.1*, with alleles from the founders Alchemy (HEX, SNP = T:T) and Claire (FAM, SNP = C:C) predicted by CIM genetic analysis to confer long and short alleles at *QFll.niab‐5A.1*, respectively. Shown are the results using template DNA from Alchemy, Claire, a 50 : 50 mix of Alchemy : Claire to create an artificial heterozygote, 10 F_5_ individuals of RIL MEL_018_2, and a negative water control. Selection of F_5_ individuals MEL_018_2_1 (Alchemy allele) and MEL_018_2_2 (Claire allele) established the *QFll.niab‐5A.1* NIL pair. (d) Subsequently, this NIL pair (*FLL5A+* and *FLL5A−*) was grown at five field trials (sites NIAB 2019, KWS 2020, NIAB 2020 and LIM 2020) and two glasshouse experiments (GH 2019 and GH 2021), where the first (flag), second and third leaves were phenotyped for length and width, and area calculated. Shown here are data for trial LIM 2021; data for all trials are shown in Fig. S4. Significant differences between each NIL line for flag leaf, leaf‐2 or leaf‐3 indicated as *, *P <* 0.05; **, *P <* 0.01; ***, *P <* 0.001, as assessed by one‐way ANOVA. Also indicated within each panel are *P‐*values for interallelic (A), interleaf (L) and allele × leaf interaction (A × L), as assessed by two‐way ANOVA. For the boxplots, vertical lines denote the median, boxes indicate the lower (25%) and upper (75%) quartiles, whiskers indicate the ranges of the minimum and maximum values, and dots predicted outliers.

**Fig. 5:**
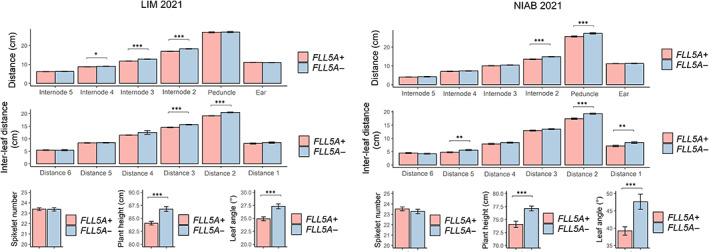
Analysis of height components, interleaf distances, flag leaf angle and spikelet number in the near‐isogenic line (NIL) pair for QTL *QFll.niab‐5A.1* (*FLL5A*). *FLL5A+* and *FLL5A−* denote the long (originating from cv Alchemy) and short (cv Claire) flag leaf length allele NIL line, respectively. Data shown are means ± SEM and were sourced from two trials (LIM 2021 and NIAB 2021), with four replicate plots per NIL line in LIM 2021 and two replicate plots per NIL line in NIAB 21, and 30 tillers harvested per plot. For interleaf distances, Distance 1 represents the distance from the base of the ear to the flag leaf, Distance 2 the distance from the flag leaf to leaf‐2, etc. Asterisks represent significant differences between the NIL pair (*, *P* < 0.05; **, *P* < 0.01; ***, *P* < 0.001) according to one‐way analysis of variance.

Located within meta‐QTL M8 on chromosome 5A, *QMFll.niab‐5A.1* (Fig. 4a,b; subsequently referred to as *FLL5A*) was selected for development of a NIL pair. Predicted *FLL5A* founder effects showed Alchemy and Xi19 carried alleles with the strongest increasing effect on flag leaf length and area, while alleles from Brompton, Claire, Rialto and Soissons had the strongest decreasing effect (Table S8). Analysis of the 90K SNP genotypic data showed RIL MEL_018_2 was heterozygous across the *FLL5A* genetic interval. Analysis of the SNP data predicted this heterozygous region to carry an allele from Alchemy vs an allele from either Brompton, Claire or Rialto (subsequently referred to here as the ‘Claire’ allele), with the predicted mean contrast in length from the genetic analysis of the MAGIC population to be 0.64 cm (Table S9). Genetic marker *BS00062996_51*, located at the QTL peak and known to be heterozygous in RIL MEL_018_2 at the F_5_ generation, was converted to a codominant KASP marker to identify sibling F_5_ MEL_018_2 individuals carrying either homozygous Alchemy (T:T; *FLL5A+*) or Claire (C:C; *FLL5A−*) genotypes. Of the 10 F_5_ individuals investigated, one was homozygous for the Alchemy allele, five homozygous for the Claire allele, and four were heterozygous (Fig. 4c). Assessment of the NIL pair in five field and two glasshouse environments found trends for increased flag leaf length combined with decreased flag leaf width for the Alchemy allele compared with that from Claire (Figs 4d, S4), with the difference being significant for length in six of the seven environments (*P <* 0.05) and for width in just the KWS 2020 trial (*P* ≤ 0.001). The trends in effects of the contrasting alleles were progressively less pronounced in leaf‐2 and leaf‐3, with significant allele differences observed for leaf‐2 length (*P =* 0.05) and width (*P <* 0.001) in the 2019 trials, for leaf‐2 and leaf‐3 width in 2020 (*P* < 0.001 and *P <* 0.05, respectively), for leaf‐2 and leaf‐3 length in both of the 2021 season trials, and for leaf‐2 in NIAB 2022. The flag leaf was shorter than leaf‐2 and leaf‐3 in all environments apart from the glasshouse 2021 experiment (GH 2021). Analysis of main tillers from the two 2021 field trials found the *FLL5A+* NIL with increased leaf length and area to also have a significantly more erect flag leaf and to have a significantly shorter plant height (*P* < 0.001; Fig. 5). This height difference manifested *via* trends of increased internode distances from the peduncle (internode‐1) all the way down to internode‐5, with significant differences observed for internode‐2 to internode‐4 in trial NIAB 2021 and for the peduncle and internode‐2 in LIM 2021. Differences in internode lengths were mirrored by those for the distances between leaves: the *FLL5A+* allele showed trends for shorter interleaf distances, with significant differences predominantly observed where the corresponding internode distance was also significantly different. However, no significant differences between *FLL5A+* and *FLL5A−* were observed for ear length or spikelet number. Based on the glasshouse 2021 experiment, there was no difference in leaf emergence rate or final leaf number between the *FLL5A* NIL pair (Fig. S5). Collectively, the Mendelization of QTL *FLL5A* (1) confirmed it as a robust source of quantitative variation in upper canopy leaf area, (2) found trade‐offs between length and width occur in some environments and (3) identified possible pleiotropic effects on flag leaf angle and internode and interleaf distances.

### Increased leaf length at FLL5A is mediated by longer epidermal pavement cells and is associated with larger stomata at lower density

**Fig. 6:**
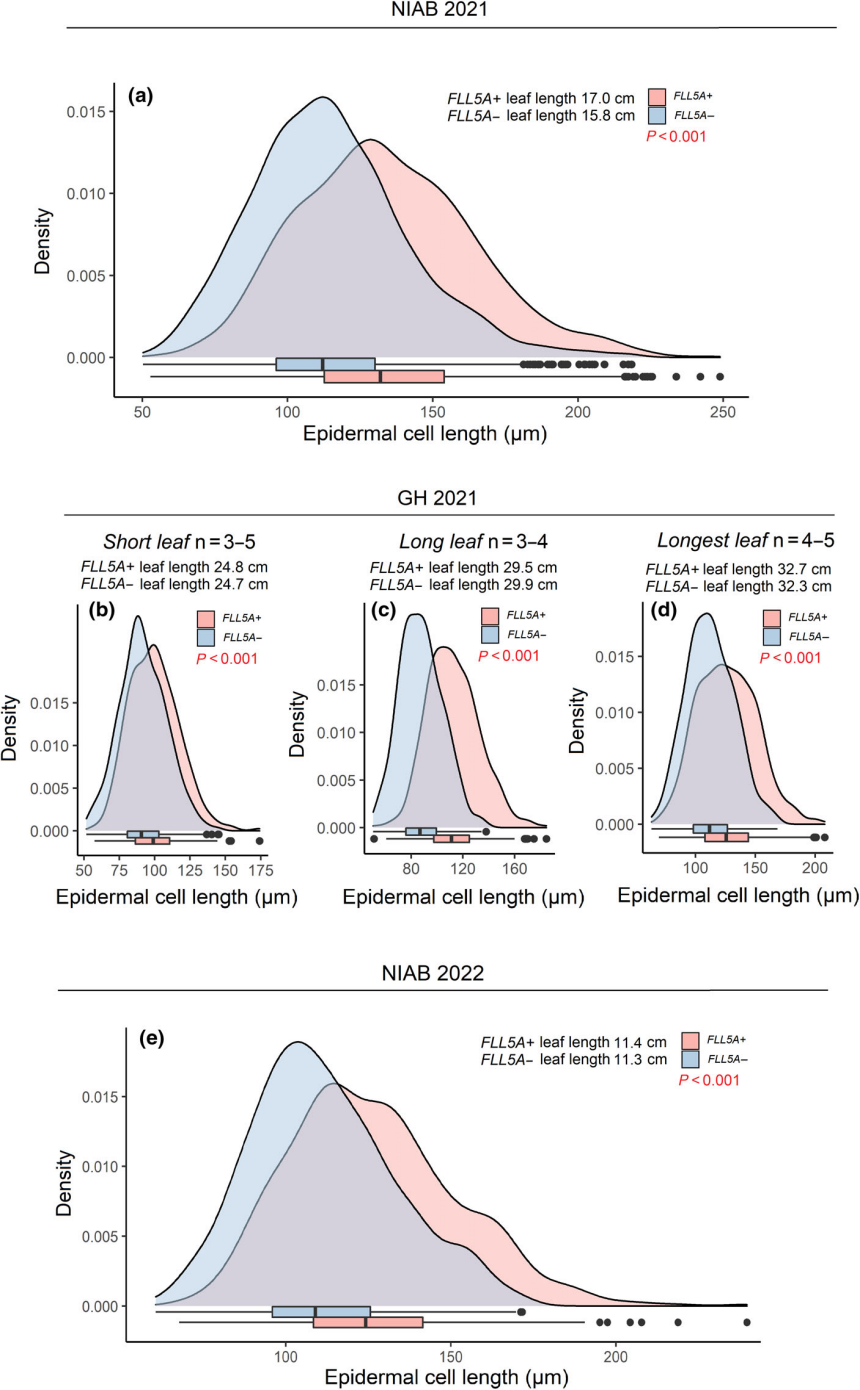
Density plots and box plots illustrating flag leaf epidermal cell length in the near‐isogenic line (NIL) pair for flag leaf morphology QTL *QFll.niab‐5A.1* (*FLL5A*). The contrasting alleles captured in the NIL pair originate from the founders Alchemy (*FLL5A+*; long flag leaf allele) and Claire (*FLL5A−*; short flag leaf allele). For boxplots, the vertical lines denote the median, boxes indicate the lower (25%) and upper (75%) quartiles, whiskers indicate the ranges of the minimum and maximum values, and dots predicted outliers. Cell length data shown are: (a) from field trial NIAB 2021, for *FLL5A+* measured from flag leaves of mean length of the *FLL5A+* plots, for *FLL5A−* plants measured from flag leaves of mean length within the *FLL5A−* plots. To determine whether difference in epidermal cell length was independent of flag leaf length, from the GH 2021 trial flag leaves of three different mean lengths were sampled for *FLL5A+* and *FLL5A−* plants: (b) 24.7 cm, (c) 29.6 cm, (d) 32.5 cm, while flag leaves of equal mean length were sampled from *FLL5A+* and *FLL5A−* plots in field trial NIAB 2022. (e) Field trial NIAB 2022, with leaves of equal length selected for each *FLL5A* allele − the mean flag leaf length of the samples studied are indicated. Cell length in all five panels were significantly different (*P* < 0.01) according to one‐way analysis of variance.

**Fig. 7:**
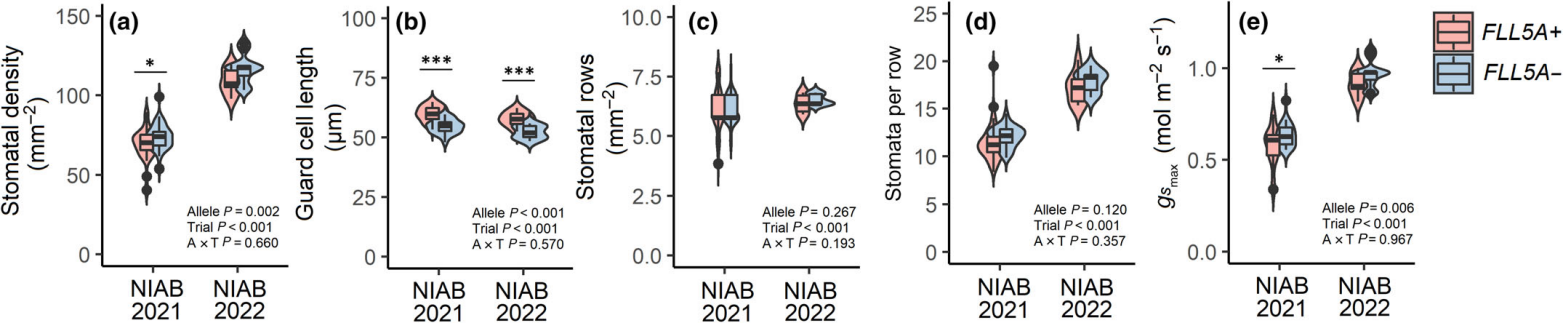
Stomata traits from the upper (adaxial) side of the mid‐region of the flag leaf for the *FLL5A* near‐isogenic line (NIL) pair grown in field trials NIAB 2021 and NIAB 2022. For NIAB 2021, flag leaves were sampled of mean size for each NIL line (*FLL5A+* = 17.0 cm; *FLL5A−* = 15.8 cm). For NIAB 2022, flag leaves of equal size were sampled from both *FLL5A+* and *FLL5A−* (11.3 ± 0.3 cm). Significant differences between each NIL line within a trial are indicated as *, *P <* 0.05; **, *P <* 0.01; ***, *P <* 0.001, as assessed by one‐way ANOVA. Also indicated within each panel are *P‐*values for interallelic (A), inter‐trial (T) and allele × trial interaction (A × T), as assessed by two‐way ANOVA. For box plots, the horizontal lines denote the median, boxes indicate the lower (25%) and upper (75%) quartiles, whiskers indicate the ranges of the minimum and maximum values, and dots predicted outliers. (a) Stomatal density, (b) guard cell length, (c) stomatal rows, (d) stomata per row, (e) *G*_smax_ (maximum stomatal conductance per unit leaf area).

To investigate whether *FLL5A* controlled contrasting flag leaf length *via* increased epidermal pavement cell size or increased cell number, we first measured pavement cell lengths in the *FLL5A* NIL pair grown at the NIAB 2021 trial. Fifteen flag leaves of average length were sampled for both the *FLL5A+* (mean = 17.0 cm) and *FLL5A−* (mean = 15.8 cm) alleles. Pavement cell lengths for *FLL5A+* were significantly longer than those for *FLL5A−* (*P* < 0.001; Fig. 6a). To determine whether the difference in pavement cell length was independent of flag leaf size *per se*, we sampled flag leaves of three sizes from each NIL line in the glasshouse GH 2021 experiment: ‘small’ (mean size for the *FLL5A−* class, 25.2 cm), ‘large’ (mean for *FLL5A+*, 30.8 cm) and ‘largest’ (mean = 32 cm). Within each leaf size class, *FLL5A+* pavement cell sizes at the middle of the flag leaf blade were significantly longer (*P* < 0.001) than those in *FLL5A−* (Fig. 6b–d). With the assumption that cell length does not change with respect to position along the leaf length, we calculated the epidermal cell number per leaf using the mean cell and leaf lengths for each NIL, finding the *c*. 20% increase in leaf length to be associated with just a *c*. 2.5% increase in cell number in the *FLL5A+* allele (*P =* 0.04, one‐way ANOVA test). While this *c*. 2.5% increase in cell number is significant, overall the results indicate *FLL5A* mediates differences in flag leaf length predominantly *via* cell length, rather than cell number. This was further supported *via* analysis of epidermal cell length in flag leaves of equal length (11.3 ± 0.3 cm) and width (1.6 ± 0.2 mm) from the NIL pair grown in field trial NIAB 2022 (Fig. 6e). Again, mean pavement cell size sampled from the centre of the flag leaf was longer in *FLL5A+* than *FLL5A−* (*P* < 0.001). Equivalent measurements at the flag leaf base and tip found that while pavement cell length was longer at the leaf tip compared the base in both NILs (*P <* 0.05), at each of the three positions, pavement cell length was significantly longer in *FLL5A+* than *FLL5A−* (*P* ≤ 0.05; Fig. S6). We next investigated whether *FLL5A*‐mediated increases in leaf size affect additional epidermal cell patterning. In both NIAB 2021 and NIAB 2022, while *FLL5A+* had longer stomata (*P <* 0.001) in the middle of the leaf, this was compensated for by lower stomatal density (significant for NIAB 2021, *P <* 0.05), most likely driven by increased stomata number per stomata row, rather than increased stomata rows *per se* (Fig. 7). This resulted in trends for higher maximum stomatal conductance per unit leaf area (*G*_smax_) in *FLL5A−* (Fig. 7). While these stomatal differences between *FLL5A+* and *FLL5A−* at the middle of the leaf were mirrored by those at the base (including *G*_smax_, *P* < 0.05), they were less apparent at the tip (Fig. S6).

### Relatively high haplotype diversity is present at the FLL5A locus in European wheat

**Fig. 8:**
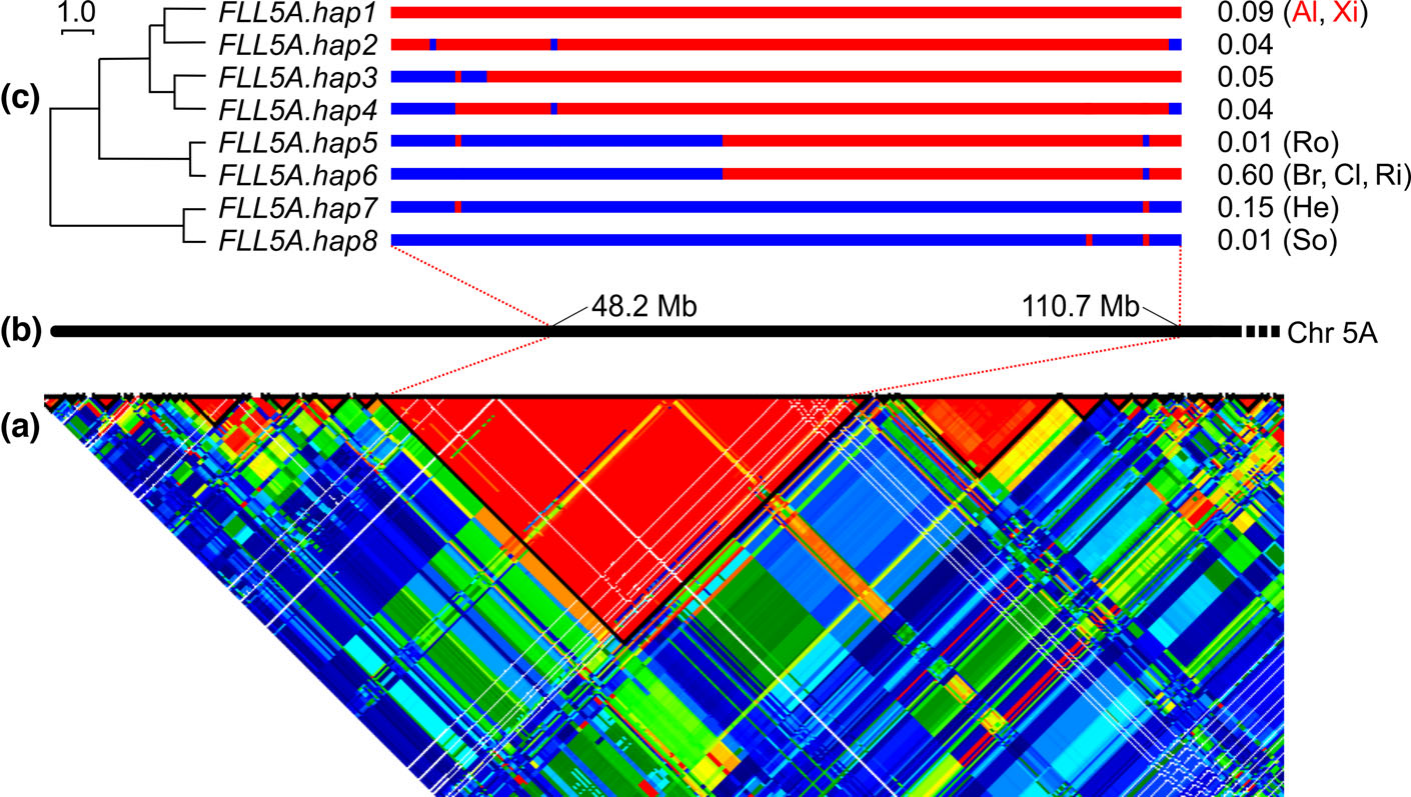
Haplotype analysis around the wheat (*Triticum aestivum* L.) genetic locus, *QFll‐niab‐5A.1* (termed, *FLL5A*). (a) Heatmap of linkage disequilibrium on a region of chromosome 5A in the panel of 403 European wheat varieties genotyped with the 90K single‐nucleotide polymorphism (SNP) array; haploblocks are highlighted *via* black lines, and the peak SNP for *FLL5A* was located in the large haploblock of 149 SNPs spanning the centromere. (b) The physical map of chromosome (Chr) 5A from the short arm telomere onwards, indicating the physical position of the manually curated subset of 125 SNPs subsequently investigated manually for defining haplotypes. (c) Haplotypes identified in the varietal panel using 125 SNPs. The Euclidian distance dendrogram to the left illustrates the relationships between haplotypes, while the frequencies of the haplotypes in the varietal panel are listed to the right. The haplotypes to which the MAGIC founders belong are indicated: Al (Alchemy), Br (Brompton), Cl (Claire), He (Hereward), Ri (Rialto), Ro (Robigus), So (Soissons), Xi (Xi19), with the founders contributing alleles with the greatest increasing effect on flag leaf length highlighted in red.

Analysis of genetic markers immediately flanking the *FLL5A* QTL peak in the eight MAGIC founders indicated more than four haplotypes were likely present (Table S9). To contextualise MAGIC founder *FLL5A* haplotypes with those present in wider wheat germplasm, we undertook haploblock analysis of the *FLL5A* locus using 403 north‐west European wheat cultivars genotyped with a 90K SNP array. Analysis of linkage disequilibrium on chromosome 5A identified 91 haploblocks (median haplotypes per haploblock = 3.0), with the haploblock containing the peak SNP from the flag leaf length meta‐analysis located between 61.59 and 65.36 cM (Fig. 8; Table S10). Subsequent analysis of this haploblock using 125 SNPs identified eight haplotypes (Fig. 8). By far, the most common in the varietal panel was haplotype *FLL5A.hap6*, found in 60% (241/403) of cultivars, including MAGIC founders Brompton, Claire and Rialto which carried a ‘short leaf’ allele at the QTL. All remaining haplotypes were present at a frequency of 15% or less. *FLL5A.hap1*, *FLL5A.hap2*, *FLL5A.hap3* and *FLL5A.hap4* formed a group of similar haplotypes present in 23% (91/403) of cultivars, with the two MAGIC founders carrying alleles with the strongest increasing effect on flag leaf length belonging to *FLL5A.hap1* (Alchemy and Xi19). Of the 15 wheat varieties for which genome assemblies are publicly available (IWGSC *et al*., 2018; Walkowiak *et al*., 2020), three were present in our variety panel: Cadenza (*FLL5A.hap1*), Robigus (*FLL5A.hap5*) and Claire (*FLL5A.hap6*). Cross‐referencing the 90K SNP data with the variants present at the corresponding genomic positions in these 15 sequenced cultivars found them to have six *FLL5A* haplotypes, two of which were new: one with most similarity to *FLL5A.hap8* identified in the two sequenced wheat varieties of Asian origin (Norin61 and Chinese Spring) and another with most similarity to *FLL5A.hap2* identified in the Australian variety LongReach Lancer and the Mexican variety Weebill1 (Table S11). As the *FLL5A* QTL interval spans, at least partially, the pericentromeric region of chromosome 5A (i.e. within a large physical region within which little genetic recombination occurs), no further analysis of gene content was undertaken.

## Discussion

Wheat canopy architecture is a key driver of source strength (the rate a plant produces photosynthetic assimilates). Relatively simple leaf morphology traits, such as length, width, area and angle, combine to create an array of different canopy architectures. We found the MAGIC founders employed contrasting flag leaf architectures to capture light. Indeed, while two of the eight founders shared broadly similar flag leaf area and angle, the components defining area differed between them: Alchemy achieved intermediate area *via* a long flag leaf of intermediate width, while Rialto flag leaves were short and wide. Thus, while differing flag leaf dimensions can lead to the same overall leaf area, questions remain as to which combinations are beneficial in which environment and how differing leaf dimensions at each layer throughout the canopy impact source strength at different stages in plant development.

By analysing a high proportion of the genetic variation employed in North‐Western European wheat germplasm captured *via* our eight‐founder population, we identified the key genetic determinants used to control flag leaf morphology within this pool. Our finding that genetic control of flag leaf size and angle traits is highly polygenic and that the loci detected often control two or more leaf traits, agrees with previous studies in other germplasm (e.g. Yan *et al*., 2020). Transgressive segregation occurred in both directions for all traits, most likely due to dispersion of contrasting alleles at multiple loci between founders (Mackay *et al*., 2020). Mendelization of our strongest flag leaf morphology QTL confirmed its effect on leaf length, area and angle and identified an effect on plant height – due either to pleiotropy or a linked gene(s). Analysis of epidermal pavement cell size in the *FLL5A* NIL pair indicated the increased leaf length observed in the *FLL5A+* NIL was predominantly due to longer cells, rather than increased cell number. This was accompanied by a more erect leaf as well as shorter plant height – due to the reduction in internode distances along the stem, rather than the reduction in the number of internodes or in the length of one or more specific internodes. Our finding that the control of leaf size and angle traits are often linked genetically suggests a common mechanism may be responsible. The process of plant cell elongation is regulated by hormones, including gibberellic acid, auxin and brassinosteroids, that stimulate cell wall relaxation, and the synthesis of new polysaccharides required for its growth (Mantilla‐Perez & Fernandez, 2017). This includes the control of cereal leaf angle, where erect leaves result from modified cell elongation on the upper or lower side of the lamina joint (Zhang *et al*., 2009; Mantilla‐Perez & Fernandez, 2017; Liu *et al*., 2019; reviewed by Luo *et al*., 2016). Modified leaf angle *via* the brassinosteroid pathway is often associated with pleiotropic effects on other traits, including plant height and leaf morphology. For example, mutations in the rice brassinosteroid receptor kinase gene *OsBRI1* lead to more erect leaves, reduced plant height (Yamamuro *et al*., 2000) and more aboveground biomass (Nakagawa *et al*., 2011). Similarly, wheat stem internode length is determined *via* cell division in the intercalary meristem (i.e. the meristem present at each node along the stem), followed by cell elongation, and again is mediated *via* phytohormones including gibberellic acid (Peng *et al*., 1999) and brassinosteroids (Gasperini *et al*., 2012). Interestingly, we found *FLL5A+‐*mediated increase in leaf size and pavement cell length was associated with larger, but less densely spaced, stomata across the flag leaf blade. Trade‐off between stomatal size and density is well established (reviewed by Bertilino *et al*., 2019). However, as smaller stomata are reported to respond more rapidly to environmental fluctuations such as light intensity, and may provide shorter diffusion pathways, they can potentially enhance photosynthetic efficiency and/or performance under drought (Ouyang *et al*., 2017). Notably, while traits relevant to flag leaf photosynthetic efficiency such as stomatal conductance, stomatal density and CO_2_ assimilation rate and are often assessed on a unit leaf area basis (e.g. Driever *et al*., 2014; Carmo‐Silva *et al*., 2017; Faralli *et al*., 2019; McAusland *et al*., 2020), such studies rarely take into account the total functional leaf area, differences between leaf layers within the canopy, or inter‐/intracultivar differences in leaf size and angle. We suggest integrating and fully exploring these considerations will lead to new insights into source strength in the context of optimising whole plant performance throughout development. The *FLL5A* NIL pairs will enable future studies on the effects of differences in leaf and stomatal morphology on whole canopy‐level photosynthetic performance and water‐use efficiency.

Whether a common gene underlies increased flag leaf size while having an epistatic effect on the other flag leaf architecture and cellular traits requires further investigation. Irrespective of whether common mechanisms and/or genes underlie the *FLL5A* trait complex, the observed longer and more erect leaf phenotype combined with shorter plant stature combines several trait combinations recognised to be part of an ideal modern wheat ideotype (Richards *et al*., 2019). This is notable, as alleles controlling desirable erect leaf phenotype in cereals are often coupled with unfavourable pleiotropic effects of other traits (Luo *et al*., 2016). Analysis of the *FLL5A* NIL pair found no difference in spikelet number per ear under field conditions, or leaf emergence rate and final leaf number under glasshouse conditions. However, further analysis is required to determine whether the *FLL5A* locus causes trade‐offs in other traits, such as tiller number, gran size, grain quality and yield. Although we identified *a priori* candidate genes within the *FLL5A* interval (wheat orthologues of the *WOX3* and *OsCsLD4* genes that control leaf size in related cereal species), this locus spans the pericentromeric region of chromosome 5A which shows very low genetic recombination (Fig. 4b). Indeed, a flag leaf width QTL has been reported at a similar location in Chinese wheat varieties within a 0.2 cM interval (Xue *et al*., 2013), corresponding to a physical interval of > 100 Mbp. Therefore, while map‐based cloning approaches to identify the gene underlying *FLL5A* may not prove possible, selection for this chromosomal region within breeding programmes will likely prove both possible and beneficial. Based on 90K SNP genotypic data in over 400 north‐west European winter wheat lines, we found a median of three haplotypes to be present per haploblock across chromosome 5A. This agrees with the limited haplotype variation thought to be present in such germplasm (Scott *et al*., 2021). However, the haploblock estimated to contain *FLL5A* contained eight haplotypes, spanning a large physical distance within the pericentromeric region. The *FLL5A* allele conferring the greatest increase in leaf length was found in MAGIC founders Alchemy and Xi19. This allele was represented by haplotype *FLL5A.hap1* in the collection of European wheats, which together with three closely related haplotypes, represented a fifth of the varieties investigated. By contrast, the most common haplotype, *FLL5A.hap6*, conferred the short leaf allele. The three MAGIC founders carrying this haplotype are all linked *via* a common grandparent (Moulin, *FLL5A.hap6*), one of the six most commonly used parents in the European wheat pedigree (Fradgley *et al*., 2019). The flag leaf trait complex associated with the *FLL5A+* allele may adapt wheat to specific target environments *via* various routes: erect leaf angles for the uppermost leaves are associated with higher yield due to increased light penetration to the lower canopy in wheat (e.g. Shearman *et al*., 2005), as well as in other cereal crops including maize (e.g. Lauer *et al*., 2012) and rice (e.g. Sinclair & Sheeny, 1999). Indeed, wheat has palisade mesophyll cells on both the upper (adaxial) and lower (abaxial) leaf surfaces, and has similar densities of stomata on both surfaces (Wall *et al*., 2022). As erect flag leaves are more likely to receive direct light to either of the leaf surfaces, the cellular characteristics of wheat should help maintain high photosynthetic carbon assimilation irrespective of which leaf surface faces the sun at any given point in the day.

While wheat genetic studies commonly focus on the flag leaf, it is the flag, second and third leaves that growers take care to keep clean from fungal infection *via* chemical control (Bouvet *et al*., 2021), and therefore are critical in building final grain yield. Analysis of our *FLL5A* NIL pair showed this locus typically increases length in the 2–3 topmost leaves, while the total leaf number per tiller and leaf emergence rate remains unchanged. Reduced plant height beyond that conferred by the semi‐dwarfing ‘Green Revolution’ genes *Rht‐B1* and *Rht‐D1* is also favourable in many agricultural environments, as plant growth regulators are commonly applied to semi‐dwarf wheat crops to further reduce height (Strydhorst *et al*., 2018). Future analysis of the *FLL5A* locus would allow further investigation of how contrasting alleles at the *FLL5A* locus impact on morphological and physiological aspects of photosynthesis, source strength, yield components, final grain yield, and how these interact with the environment over the plant lifecycle. Furthermore, creating near‐isogenic germplasm which combine contrasting alleles at two or more genetic loci will help further explore the trade‐offs between source and sink traits. For example, a relatively large effect grain size QTL *Qtgwcb‐5A* is present in European germplasm *c*. 10 cM from *FLL5A*, with increased grain size associated with longer grain pericarp cell length (Brinton *et al*., 2017). By combining alleles at QTL controlling increased source strength with those increasing sink strength, it should be possible to gain further insights into the design of improved crop ideotypes, and how these perform in different agricultural environments.

### Concluding remarks

We demonstrated that: (1) the use of different canopy architectures to capture light is a common strategy in wheat, (2) genetic control of wheat flag leaf size traits is highly polygenic, (3) *FLL5A* is an important genetic determinant of multiple wheat canopy architecture traits, (4) *FLL5A* mediates longer leaf length *via* increased epidermal cell length, (5) these increases are associated with fewer but larger stomata, and (6) the haplotype capturing the *FLL5A+* long leaf allele is relatively common, but not predominant, in North‐Western European wheat varieties released over the last 70 yr. This work highlights the need to establish how different canopy architecture strategies deployed at different layers of the canopy affect source strength under different environmental conditions and plant growth stages, and the need to accurately incorporate leaf size into measurement of photosynthesis‐relevant traits commonly measured on a per unit area basis.

## Competing interests

None declared.

## Author contributions

CM‐B, MR, RD and JC undertook field phenotyping of the population. GM, MR, CM‐B, BC, CMZ and JC analysed the resulting phenotypic data. CMZ, GM, MR and CM‐B undertook genetic analyses. ASR, CM‐B and JC developed and genotyped NILs. JJ, SB and JC grew NILs. ASR, GC and JC phenotyped NILs. MF and JC analysed NIL data. CMZ and JC undertook haplotype analysis. AG, KAG, JJ, TL, ESO and JVR provided project resources and support. BC, TL and JC provided project supervision. JC wrote the manuscript, with inputs from CMZ, GM and BC. All authors edited and approved the manuscript. CMZ, MR, CM‐B, GM and BC contributed equally to this work.

## Supporting information


**Fig. S1** Example stems of the eight MAGIC founders at anthesis.
**Fig. S2** Histograms of flag leaf phenotypic data (best linear unbiased estimated) collected from trials in seasons 2016, 2017, 2018 and 2019.
**Fig. S3** Gene expression data for the *a priori* candidate genes *TaSMOS1‐D*, *TaBU1‐A*, *TaWOX3‐A* and *TaCsLD4‐A*.
**Fig. S4** Leaf size data for the uppermost three leaves in the *QFll.niab‐5A.1* (*FLL5A*) near‐isogenic line pair assessed at all five field sites and two glasshouse experiments.
**Fig. S5** Leaf emergence rate for the flag leaf QTL *QFll.niab‐5A.1* (*FLL5A*) near‐isogenic line pair.
**Fig. S6** Flag leaf epidermal cell and structure phenotypes in the *FLL5A* NIL pair grown at the NIAB 2022 field trial.
**Methods S1** Supplementary text for the Materials and Methods and Results sections.

**Table S1** Agronomy packages used in the 2016, 2017, 2018 and 2019 season MAGIC field trials.
**Table S2** Details of the NIAB Elite MAGIC field trial details undertaken to study flag leaf traits.
**Table S3** Primer details for Kompetitive Allele‐Specific PCR marker *BS00062996_51* on chromosome 5A.
**Table S4** Details of the five field trials undertaken for the near‐isogenic line pair developed for the flag leaf QTL *QFll.niab‐5A.1* (*FLL5A*) and the number of culms per plot assessed for flag leaf length morphometric traits.
**Table S5** Flag leaf phenotypic data. Best linear unbiased predictors for the four field trials (columns C to S) and for the meta‐analysis (columns).
**Table S6** Flag leaf traits for the eight NIAB Elite MAGIC founders from the field trials conducted in the UK in harvest seasons 2016, 2017, 2018 and 2019.
**Table S7** Genetic mapping results for flag leaf area, length, width, length : width ratio and angle of the NIAB Elite MAGIC population at four field trials.
**Table S8** Candidate genes, based on wheat orthologues of cloned leaf size or angle genes from related cereal species (rice, maize and barley).
**Table S9** Chromosome 5A 90K single‐nucleotide polymorphism array genotypic data for MAGIC recombinant inbred line MEL_018_2 and the eight MAGIC founders.
**Table S10** 90K SNP genotype calls for all of chromosome 5A in the European variety panel (*n* = 403).
**Table S11** Haplotypes at QTL for flag leaf length (*QFll.niab‐5A.1*) and flag leaf length : width ratio (*QLwr.niab‐5A*.1).
Please note: Wiley is not responsible for the content or functionality of any Supporting Information supplied by the authors. Any queries (other than missing material) should be directed to the *New Phytologist* Central Office.

## Data Availability

All phenotypic data are provided in the [Link], [Link]. MAGIC genotypic data are published previously.
