# Supplementary material for: Longer epidermal cells underlie a quantitative source of variation in wheat flag leaf size
Source: New Phytol. 2022 Dec 28;237(5):1558–73. doi: 10.1111/nph.18676 (PMC10107444; doi:10.1111/nph.18676)
Supplement: Supplementary file 1 — Fig. S1 Example stems of the eight MAGIC founders at anthesis. Fig. S2 Histograms of flag leaf phenotypic data (best linear unbiased estimated) collected from trials in seasons 2016, 2017, 2018 and 2019. Fig. S3 Gene expression data for the a priori candidate genes TaSMOS1‐D, TaBU1‐A, TaWOX3‐A and TaCsLD4‐A. Fig. S4 Leaf size data for the uppermost three leaves in the QFll.niab‐5A.1 (FLL5A) near‐isogenic line pair assessed at all five field sites and two glasshouse experiments. Fig. S5 Leaf emergence rate for the flag leaf QTL QFll.niab‐5A.1 (FLL5A) near‐isogenic line pair. Fig. S6 Flag leaf epidermal cell and structure phenotypes in the FLL5A NIL pair grown at the NIAB 2022 field trial. Methods S1 Supplementary text for the Materials and Methods and Results sections. [file NPH-237-1558-s001.pdf]

New Phytologist

***New Phytologist* Supporting Information**

Article title: Longer epidermal cells underlie a quantitative source of variation for wheat flag leaf size

Authors: Camila M. Zanella, Marilena Rotondo, Charlie McCormick-Barnes, Greg Mellers, Beatrice Corsi, Simon Berry, Giulia Ciccone, Rob Day, Michele Faralli, Alexander Galle, Keith A. Gardner, John Jacobs, Eric S. Ober, Ana Sánchez del Rio, Jeroen Van Rie, Tracy Lawson, James Cockram

Article acceptance date: 17 November 2022

The following Supporting Information is available for this article:

Fig. S1 Example stems of the eight MAGIC founders at anthesis.

Fig. S2 Histograms of flag leaf phenotypic data (best linear unbiased estimated) collected from trials in seasons 2016, 2017, 2018 and 2019.

Fig. S3 Gene expression data for the *a priori* candidate genes *TaSMOS1-D, TaBU1-A, TaWOX3-A* and *TaCsLD4-A*.

Fig. S4 Leaf size data for the uppermost three leaves in the *QFll.niab-5A.1* (*FLL5A*) near isogenic line pair assessed at all five field sites and two glasshouse experiments.

Fig. S5 Leaf emergence rate for the flag leaf QTL *QFll.niab-5A.1* (*FLL5A*) near isogenic line pair.

Fig. S6 Flag leaf epidermal cell and structure phenotypes in the *QFll.niab-5A.1* (*FLL5A*) NIL pair grown at the NIAB 2022 field trial.

Table S1 Agronomy packages used in the 2016, 2017, 2018 and 2019 season MAGIC field trials.

Table S2 Details of the NIAB Elite MAGIC field trial details undertaken to study flag leaf traits.

Table S3 Primer details for KASP marker *BS00062996_51* on chromosome 5A.

Table S4 Details of the five field trials undertaken for the nearly isogenic line (NIL) pair developed for the flag leaf QTL *QFll.niab-5A.1* (*FLL5A*), and the number of culms per plot assessed for flag leaf length morphometric traits.

Table S5 Flag leaf phenotypic data. BLUPs for the four field trials (columns C to S) and for the meta-analysis (columns).

Table S6 Flag leaf traits for the eight NIAB Elite MAGIC founders from the field trials conducted in the UK in harvest seasons 2016, 2017, 2018 and 2019.

Table S7 Genetic mapping results for flag leaf area, length, width, length:width ratio and angle of the NIAB Elite MAGIC population at four field trials.

Table S8 Candidate genes, based on wheat orthologues of cloned leaf size or angle genes from related cereal species (rice, maize, barley).

Table S9 Chromosome 5A 90k single nucleotide polymorphism (SNP) array genotypic data for MAGIC recombinant inbred line MEL_018_2, and the eight MAGIC founders.

Table S10 90k SNP genotype calls for all of chromosome 5A in the European variety panel (n=403).

Table S11 Haplotypes at QTL for flag leaf length (*QFll.niab-5A.1*) and flag leaf length/width ratio (*QLwr.niab-5A.*1).

Methods S1 Supplementary text for the Materials and Methods and Results sections.

Fig. S1 Example stems of the eight MAGIC founders at anthesis: Alchemy (Al), Brompton (Br), Claire (Cl), Hereward (He), Rialto (Ri), Robigus (Ro), Soissons (So), Xi19 (Xi). Materials were sourced from 2022 season 1x1 m nursery plots grown at Duxford, UK.

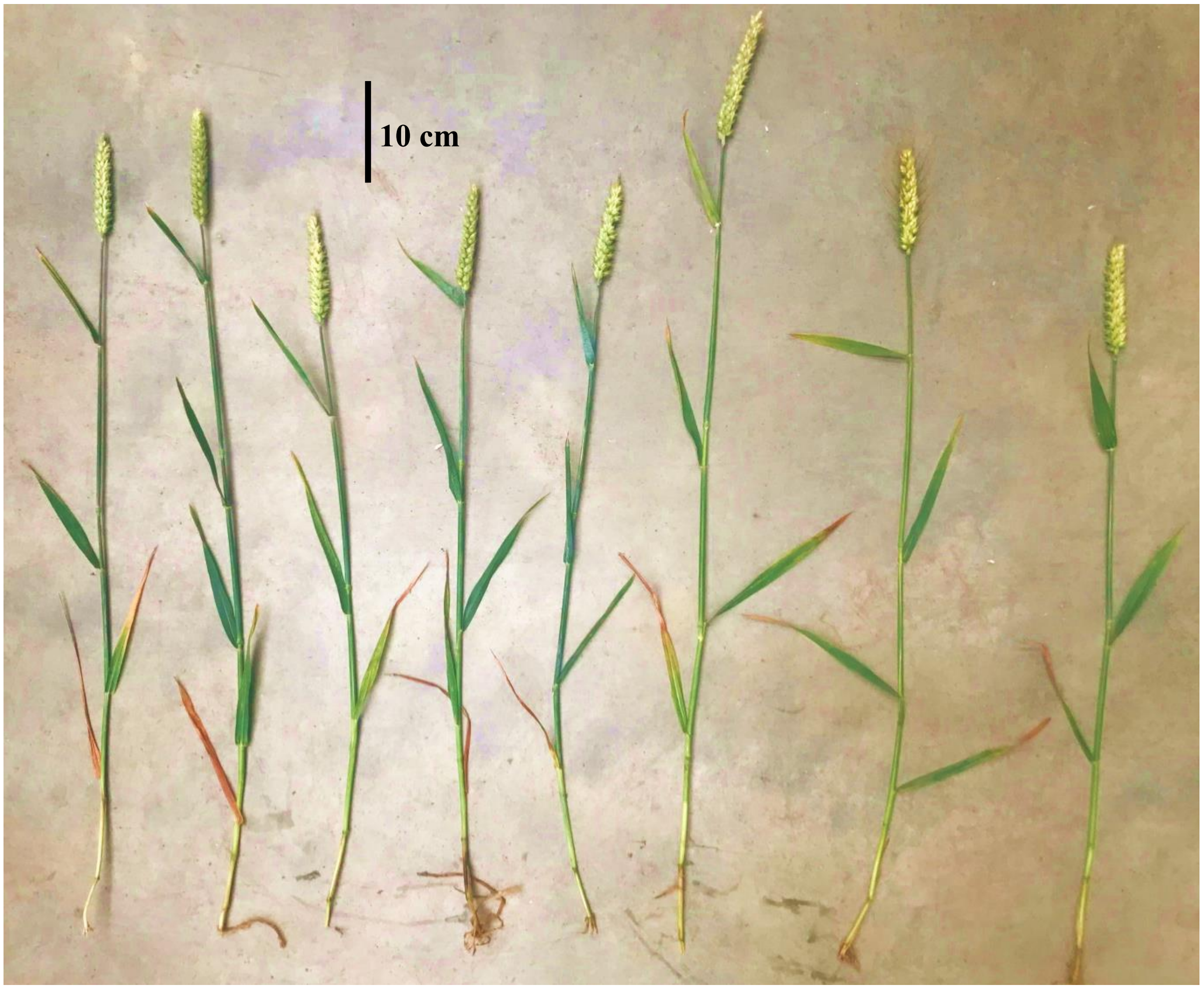

Al Br Cl He Ri Ro So Xi

Fig. S2 Histograms of flag leaf phenotypic data (best linear unbiased estimated) collected from trials in seasons 2016, 2017, 2018 and 2019. The phenotypic values of the MAGIC founders with the upper- and lower-most score for each trait are indicated by the dashed vertical lines, colour coded as shown in the key. An issue with the flag leaf width data from 2016 was identified, and so width and length:width ratio data for 2016 are not presented here.

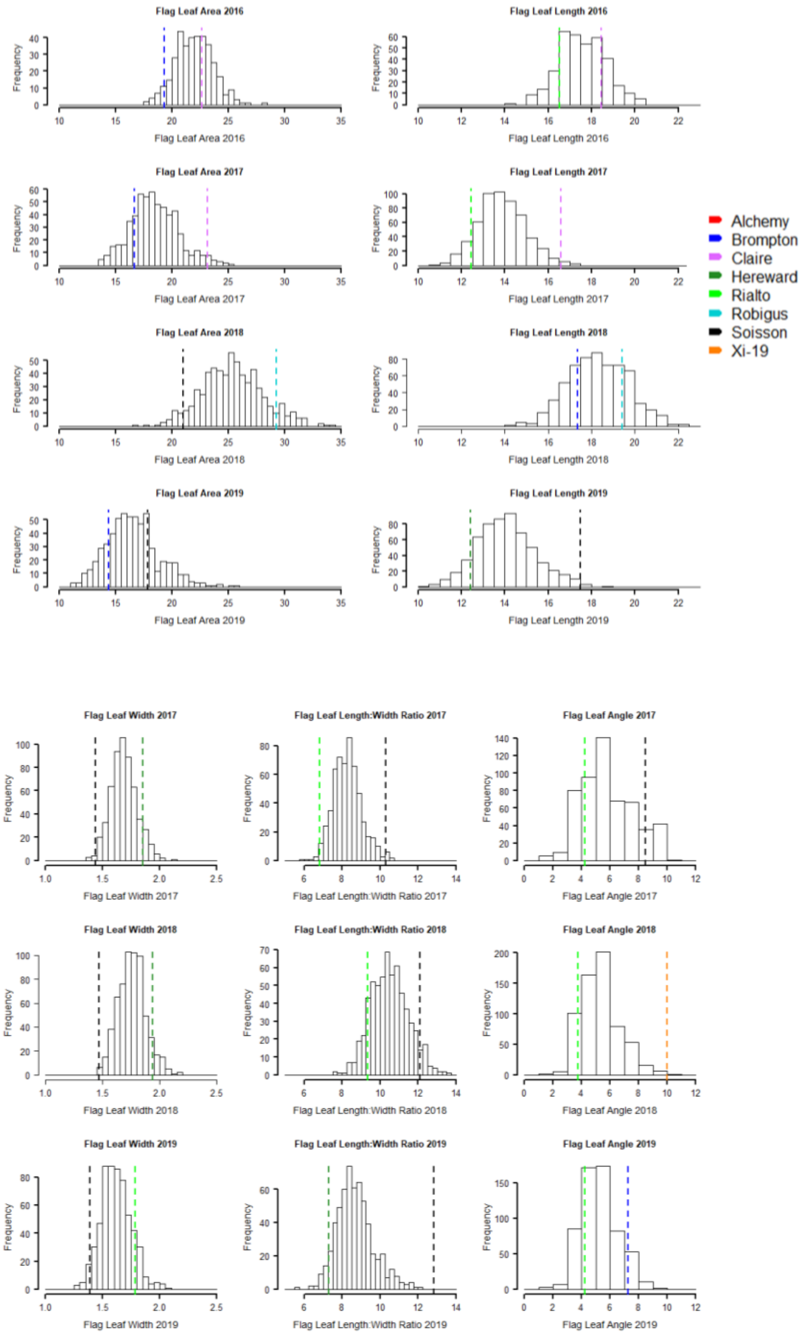

Fig. S3 Gene expression profile across 71 tissue/developmental stage combinations of *a priori* candidate genes located within wheat MAGIC flag leaf size/angle QTL intervals. Data is for wheat variety Azhurnaya, sourced from eFP Browser at https://bar.utoronto.ca/. Error bars denote ± 1 standard deviation.

TraesCS1D02G242800 (*TaSMOS1-D*)

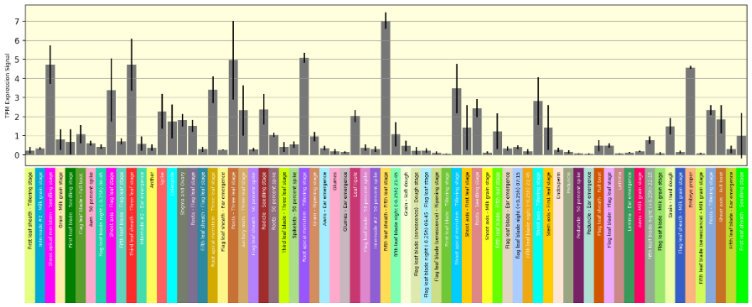

TraesCS5A02G090500 (*TaCslD4-A*)

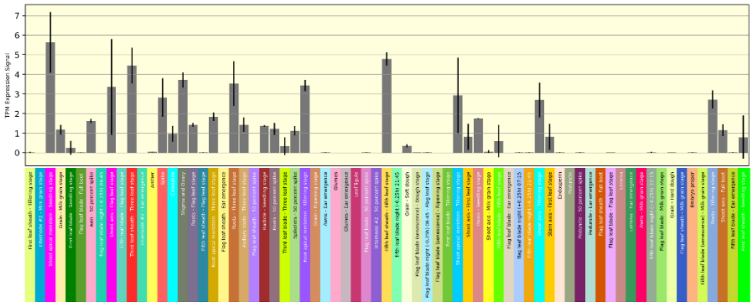

TraesCS5A02G157300 (*TaNL2-A/TaNL3-A/TaNS1-A/TaNS2-A*)

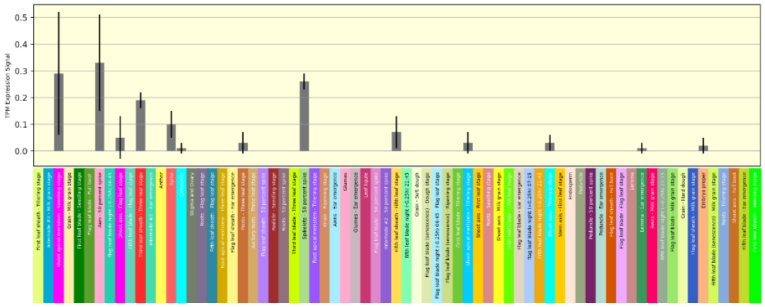

TraesCS5A02G292900 (*TaBC1-A*)

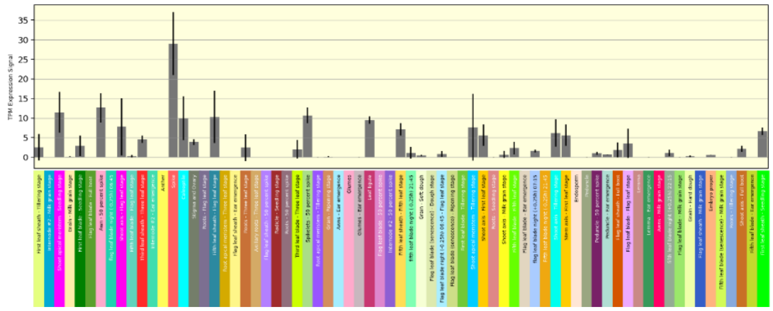

Fig. S4 Leaf size data for the uppermost three leaves in the *QFll.niab-5A.1* (*FLL5A*) near isogenic line pair assessed at all five field sites (NIAB 2019, KWS 2020, NIAB 2021, LIM 2021, NIAB 2022) and two glasshouse experiments (GH 2019, GH 2021). *FLL5A+* = long leaf allele; *FLL5A-* = short leaf allele. First (flag), second and third leaves were phenotyped for length and width, and area calculated as described in the Materials and Methods. Significant differences between each NIL line for leaf-1, leaf-2 or leaf-3 indicated as $p<0.05^{*}$, $p<0.01^{**}$, $p<0.001^{***}$, as assessed by one-way ANOVA. Also indicated within each panel are *p*-values for inter-allelic (A), inter-leaf (L), and allele x leaf interaction (A x L), as assessed by two-way ANOVA. For the boxplots, horizontal lines denote the median, boxes indicate the lower (25%) and upper (75%) quartiles, whiskers the ranges of the minimum and maximum values, and dots predicted outliers.

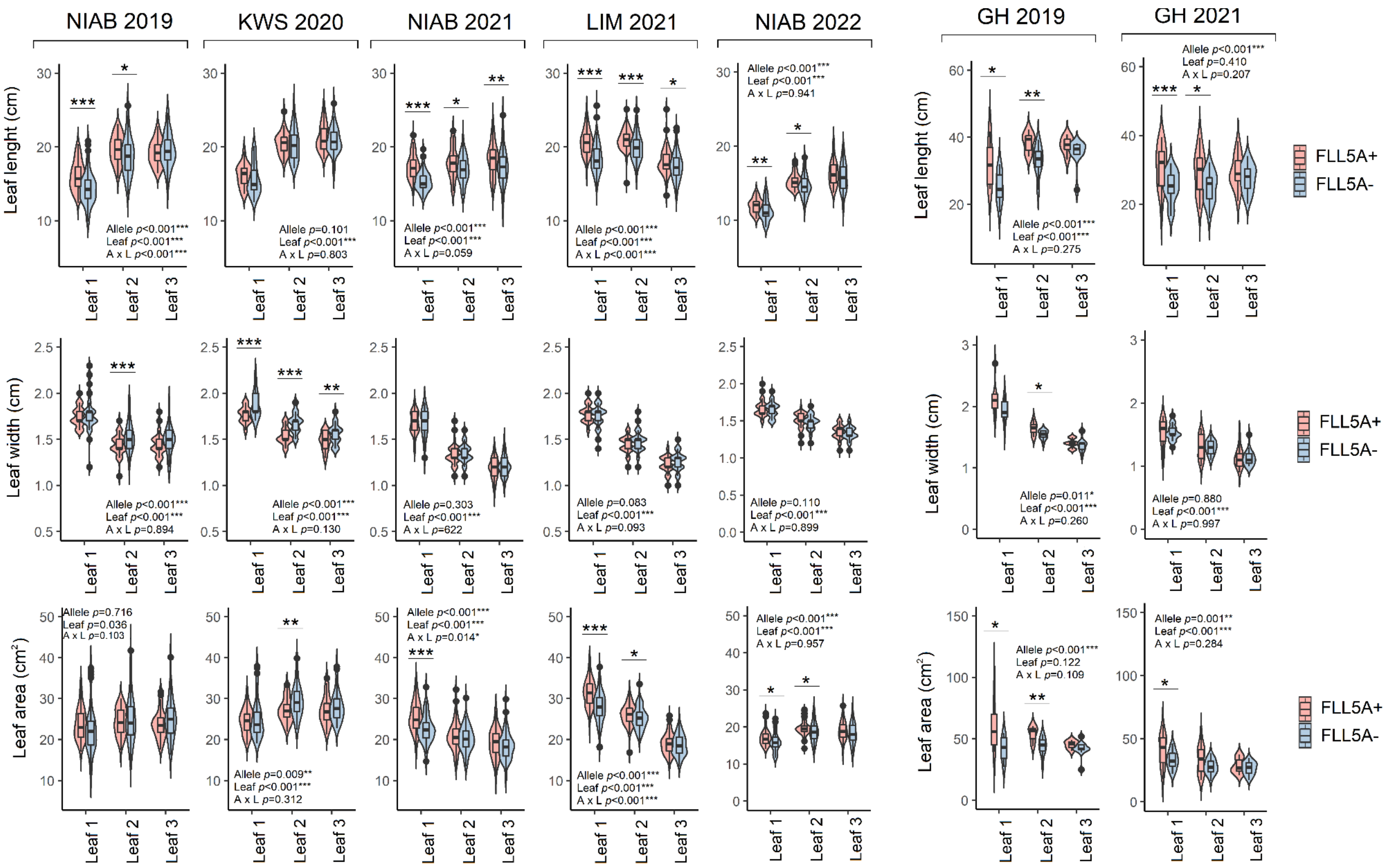

Fig. S5 Leaf emergence rate for the flag leaf QTL *QFll.niab-5A.1* (*FLL5A*) near isogenic line pair. Large leaf allele = *FLL5A+* (number of plants = 22); short leaf allele = *FLL5A-* (number of plants = 19). The x-axis shows days after transplant of vernalized seedlings to glasshouse growth conditions. At each time-point displayed, mean ± 1 standard deviation is shown. Trial = glasshouse experiment in 2021 (GH 2021). For both lines, final leaf number was found to be 10.

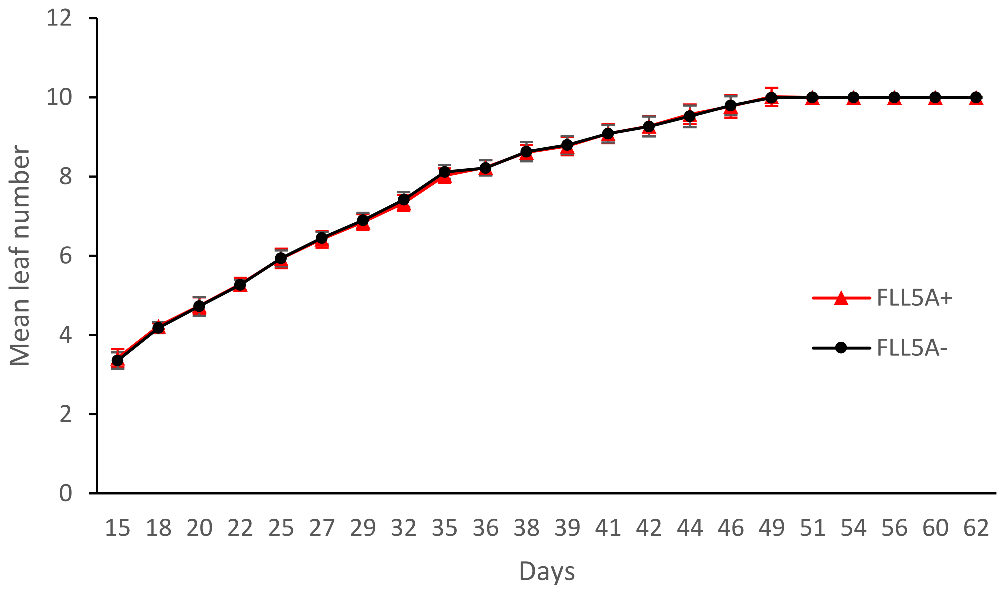

Fig. S6 Flag leaf epidermal cell and structure phenotypes in the *QFll.niab-5A.1* (*FLL5A*) NIL pair grown at the NIAB 2022 field trial. *FLL5A+* = long leaf allele; *FLL5A-* = short leaf allele. Phenotypes were determined from epidermal imprints taken from three regions of the upper (adaxial) side of the flag leaf blade: base, middle and top. For each position, four traits were phenotyped: (a) epidermal pavement cell length, (b) stomata density mm^{-2}, (c) guard cell length, and (d) stomata rows mm^{-2}. This allowed calculation of two additional traits: (e) number of stomata per stomata row mm^{-2} and (f) maximum stomatal conductance (G_{smax}). Significant differences between each NIL line for data as measured from each of the three flag leaf positions are indicated as p<0.05*, p<0.01**, p<0.001***, as assessed by one-way ANOVA. Similarly, significant differences between leaf positions are shown (in red). Also indicated within each panel are p-values for inter-allelic (A), inter-leaf (L), and allele x leaf interaction (A x L), as assessed by two-way ANOVA. For the boxplots, horizontal lines denote the median, boxes indicate the lower (25%) and upper (75%) quartiles, whiskers the ranges of the minimum and maximum values, and dots predicted outliers.

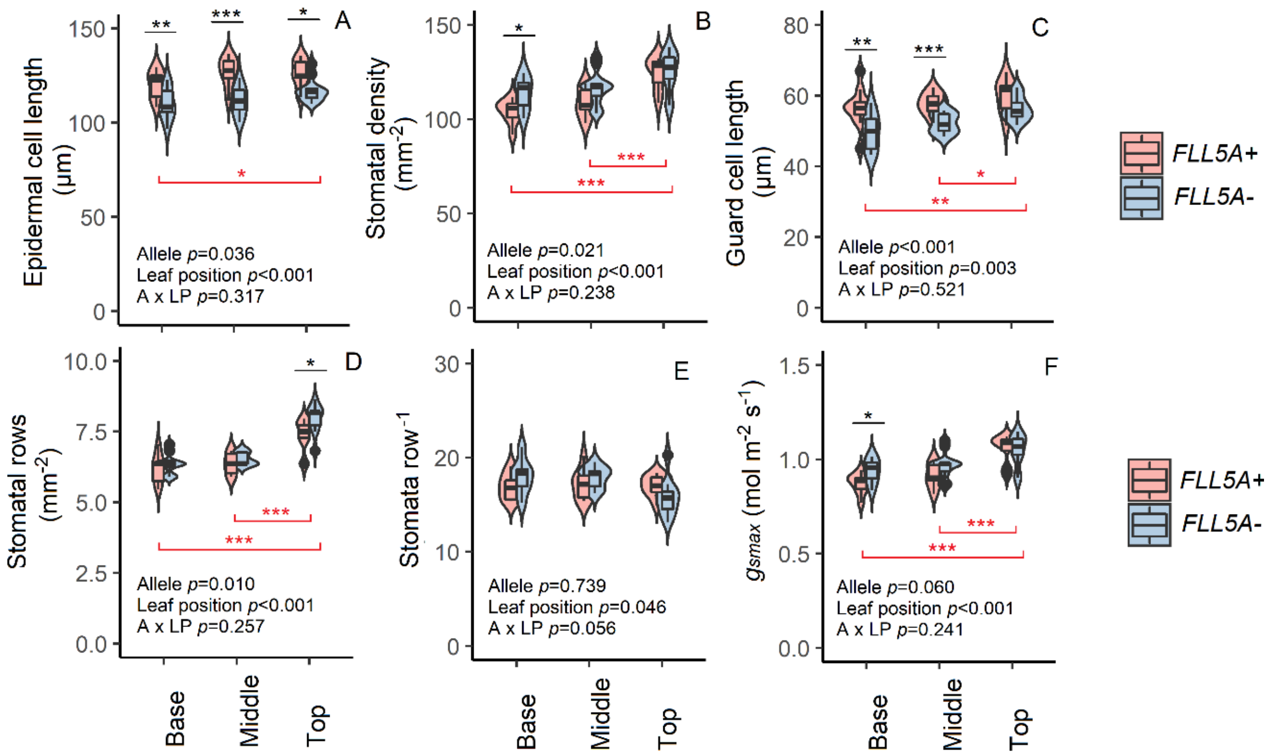

Tables S1-S11 Tables S1-S11 provided in separate Excel file.

Methods S1 Supplementary text for the Materials and Methods and Results sections.

Methods

NIAB Elite MAGIC population trial design methods

The 2016 and 2017 NIAB Elite MAGIC trials followed a p-rep design, consisting of the 8 MAGIC founders and the control variety KWS Santiago (each in 4 replicate plots per line), and 770 MAGIC RILs in 2016 (496 in 2 replicates and 274 in 1 replicate) and 770 RILs in 2017 (514 in 2 replicates and 256 in 1 replicate). The 2018 and 2019 trials each consisted of the eight founders and one control variety in 4 replicates per line, plus 700 MAGIC RILs in 2 replicates per line.

Genetic analysis methods

For IBS and IBD, multiple-test correction was carried out using the 'p.adjust' function in R, with a threshold of $p<0.05$. For IM and CIM an empirical p threshold of 0.05 was determined for each trait by conducting 100 simulations, using the R function 'sim.sigthr'. This value, together with a window size of 100 markers, was used to determine QTL peaks using the function 'find.qtl'. All detected QTL were fitted in a full model using the function 'fit.qtl', and QTL retained where $p<0.05$ and the percentage variance explained was >1% in the fitted model. The full model including all detected QTL was fitted to estimate the overall phenotypic variance explained (R^2) per trait. QTL were called based on the results of IM, providing the significance values and percentage variance explained for all QTL reported here. IBS and IBD was undertaken to further confirm QTL detected by IM. In cases where QTL were detected using both IM and CIM, QTL intervals were defined by CIM-cov10 or CIM-cov5; otherwise, intervals were defined by IM.

KASP genetic marker validation methods

KASP marker *BS00062996_51* was validated using DNA from each of the eight founders extracted from leaf material using a Plant Mini Kit (Qiagen). KASP genotyping was undertaken as described by Downie *et al*. (2018) using three technical replicates per founder. Additionally, a

no-template negative control was assessed, as well as a 50:50 mix of DNA from two founders known to contrast for allelic state to create an artificial heterozygote at the target SNP.

Scanning electron microscopy methods

Leaf sections were plunge-frozen in liquid nitrogen-cooled ethane and freeze-dried using a liquid-nitrogen cooled turbo freeze drier (Quorum K775X). Samples were mounted on aluminium SEM stubs using double-sided sticky tape and silver paint and were sputter coated with 35 nm gold using a Quorum K575X sputter coater. Samples were viewed using a Verios 460 scanning electron microscope (FEI/Thermo Fisher Scientific) run at 30 keV and 3.2 nA using the concentric backscatter detector in field-free mode.

NIL germplasm development methods

Comparison of the patterns homozygous and heterozygous genotypic calls for SNPs spanning the QTL with the corresponding allele calls in the eight founders allowed identification of the founders contributing to the observed heterozygous chromosomal regions. The donor founders for each of these RILs were cross-referenced with the predicted founder allelic affects at the 5A QTL, as determined by CIM analysis. Accordingly, RILs heterozygous across the target QTL were prioritised based on: (i) presence of founder alleles predicted to maximise phenotypic contrast, and (ii) minimising the genetic/physical interval heterozygous across the target QTL.

NIL field growth and phenotyping methods

NIL trials followed standard local agronomic practices, including full fungicide and pesticide treatment.

NIL glasshouse experiment growth methods

In the 2018 glasshouse experiment (GH 2018), twelve replicates for each of the four NIL germplasm stocks, as well as twelve replicates for each MAGIC founder, were randomised into 2 main blocks and 19 sub-blocks. In the 2021 glasshouse experiment (GH 2021), 22 replicates of each of the two lines were sown across two main blocks, with four sub-blocks per main block. For both trials, entries were sown into 96-well trays, and vernalized for 10 weeks (4 °C, 12 hour photoperiod), after which seedlings were transplanted into 1l compost filled pots and grown under temperature-controlled glasshouse conditions (24 °C day, min 15 °C night, ± 2 °C), with inductive photoperiods (16 hours light, 8 hours dark) maintained using supplementary lighting.

Results

A priori candidate gene results

Wheat homologue of rice leaf angle gene *SMOS1* was located within flag leaf angle QTL *QAng.niab-1D.1*. Wheat homologue of rice leaf angle gene *OsBU1* was located within the QTL controlling flag leaf length, area, width and angle on chromosome 7A (*QFll.niab-7A.1, QFla.niab-7A.1, QFlw.niab-7A.1, QAng.niab-7A.1*). Finally, wheat homologues of cereal genes encoding WOX3 proteins controlling leaf size in rice (*nl2, nl3*) and maize (*ns1*, *ns2*), as well as homologues of the rice leaf size gene *OsCsLD4*, were identified within the chromosome 5A pericentromeric multi-trait genetic locus M8 controlling wheat flag leaf length, width, area and length. Some aspects of gene expression profiles as determined in the wheat gene expression atlas were broadly similar for all four genes, with strong expression in the shoot apical meristem (which included the leaf primordia) at the first leaf stage as well as in the spike at full boot stage (Fig. **S3**). Of note, three of the genes (*TaSMOS1-D*, *TaCslD4-A* and *TaNRL1-A*) also showed increased expression in the shoot apical meristem (which included the leaf primordia) at the tillering sage, in the shoot axis at the flag leaf stage, and in the ovary at anthesis. *TaSMOS1-D* and *TaCslD4-A* also showed increased expression in the roots and root meristem at the tillering stage. Remaining wheat homologues of cloned cereal leaf size/angle genes were located within QTL identified in a single year only, and are not discussed.

References

Downie RC, Bouvet L, Furuki E, Gosman N, Gardner KA, Mackay IJ, Mantello CC, Mellers G, Phan HTT, Rose GA et al. 2018. Assessing European wheat sensitivities to *Parastagonospora nodorum* necrotrophic effectors and fine-mapping of the *Snn3-B1* locus conferring sensitivity to the effector SnTox3. *Frontiers in Plant Science* **9:** 881.
